# Sijunzi San alleviates the negative energy balance in postpartum dairy cows by regulating rumen fermentation capacity

**DOI:** 10.3389/fvets.2024.1512081

**Published:** 2024-12-18

**Authors:** Feifei Wang, Yongxia Mao, Chenlei Li, Yanfen Ma, Yansheng Guo

**Affiliations:** ^1^College of Animal Science and Technology, Ningxia University, Yinchuan, China; ^2^Key Laboratory of Ruminant Molecular and Cellular Breeding of Ningxia Hui Autonomous Region, College of Animal Science and Technology, Ningxia University, Yinchuan, China

**Keywords:** Sijunzi San, rumen fermentation, rumen microorganisms, lipid metabolism, negative energy balance

## Abstract

**Introduction:**

Postpartum dairy cows are susceptible to negative energy balance caused by decreased feed intake and the initiation of lactation. Sijunzi San, a famous Chinese traditional herbal formulation, can promote gastrointestinal digestion and absorption and improve disorders of intestinal microbiota. Therefore, we hypothesized that Sijunzi San might alleviate negative energy balance in postpartum dairy cows by modulating the structure of the rumen microbiota and enhancing its fermentation capacity.

**Methods:**

Liquid chromatography-mass spectrometry (LC–MS/MS) was utilized *in vitro* to identify the main active ingredients in the Sijunzi San. Techniques including *in vitro* ruminal fermentation, gas chromatography, and 16S rRNA high-throughput sequencing were employed to evaluate their effects on the structure of the rumen microbiota. To test their *in vivo* effects, sixteen postpartum Holstein dairy cows, with similar body condition and parity, were randomly assigned to two groups, with 8 cows per group. The CONT group was fed a basic diet, while the SJZS group received an additional 300 g/d of Sijunzi San along with the basic diet, continuously for 7 days. ELISA and untargeted metabolomics using ultra-high-performance liquid chromatography-tandem mass (UHPLC–MS/MS) were employed to assess the impacts on immunoglobulin levels, fat mobilization, and the blood metabolome in postpartum dairy cows.

**Results:**

Doses of 100 to 500 mg of the Sijunzi San significantly enhanced gas production, microbial protein (MCP), and short-chain fatty acid (SCFA) levels, while notably reducing pH and NH_3_-N content (*p* < 0.05), exhibiting a significant dose-dependent relationship. The results revealed that 500 mg of the prescription significantly increased the abundances of the *Succiniclasticum* and *Prevotella* genera and notably decreased the abundances of the *Christensenellaceae_R-7_group*, *Muribaculaceae*, *UCG-005*, *Comamonas*, and *F082* genera (*p* < 0.05). *Succiniclasticum* and *Prevotella* showed a significant positive correlation with ruminal SCFAs, whereas *UCG-005* exhibited a significant negative correlation with them (*p* < 0.05). Additionally, Luteolin and Glycitein were significantly positively correlated with *Prevotella*, while Licochalcone B and Liquoric acid were significantly negatively correlated with *Comamonas* (*p* < 0.05). Subsequently, the prescription significantly increased the concentrations of IgA, IgM, and microsomal triglyceride transfer protein (MTTP) in the blood (*p* < 0.01), while reducing the levels of ketones (KET) (*p* < 0.05), non-esterified fatty acids (NEFA), and triglycerides (TG) (*p* < 0.01). Notable alterations were observed in 21 metabolites in the blood metabolome (*p* < 0.05). Additionally, metabolic pathways associated with linoleic acid metabolism and steroid hormone biosynthesis were significantly affected.

**Discussion:**

The findings suggest that administering Sijunzi San to dairy cows during the postpartum period can ameliorate negative energy balance by stimulating rumen fermentation and modifying the composition and abundance of the rumen microbiota.

## 1 Introduction

With the progression of modern dairy farming methods, global milk output is witnessing a sustained increase (1). Nevertheless, this increase poses significant risks to the postpartum health of dairy cows (2). After parturition, dairy cows experience stress, reduced feed intake, and increased energy demands for lactation, leading to physiological changes such as negative energy balance (NEB), endocrine imbalances, altered ruminal fermentation, and immune suppression (3, 4). NEB can result in metabolic disorders like ketosis and fatty liver, which not only lower milk yield but also shorten cow lifespan, negatively affecting farm profitability (5). The ruminal microbiota and its fermentation function are pivotal in sustaining the health and ensuring the efficiency of dairy cow production (6). Research has shown that alterations in the composition of the ruminal microbiota and reductions in fermentation efficiency in postpartum dairy cows exacerbate NEB (7). Consequently, ruminal microbiota is crucial to the energy homeostasis, metabolic state, and physiological adaptation of postpartum dairy cows (8).

Traditional Chinese Medicines (TCMs) and their extracts are increasingly being explored as additives in animal production, due to their potential benefits for animal health, disease prevention, and overall performance. Recent studies highlight the positive effects of specific TCMs on ruminants’ production performance and health. For instance, Rahman et al. (9) found that the addition of plantain and lemongrass not only elevated feed intake and milk production, but also boosted immune function and preserved liver health in dairy cows. Antonius et al. (10) discovered that the addition of Eurydome longifolia and *Cassia alata* effectively improved rumen fermentation and reduced methane emissions in ruminants. Based on multi-omics studies, Yi et al. (11) found that the moderate supplementation of peppermint extract to the diet of fattening sheep enhanced intestinal digestion, increased the rumen abundance of beneficial bacteria, and regulated blood metabolism, thereby enhancing the growth performance of fattening sheep. Ma et al. (12) demonstrated that honeysuckle extract could alleviate heat stress in dairy cows by improving antioxidant capacity, maintaining endocrine homeostasis, and enhancing immune function. While existing studies have underscored the potential benefits of individual TCM herbs, there has been comparatively limited exploration into the application of Chinese traditional herbal formulation, combinations of multiple herbs with synergistic effects, in the context of animal production.

In traditional Chinese veterinary medicine, the rumen is considered a part of the spleen system, which plays a key role in digestion and metabolic regulation. Sijunzi San, a classic prescription for treating spleen deficiency syndrome, is composed of Ginseng, Atractylodes macrocephala, Poria cocos, and Licorice, which have effects of replenishing *qi* and strengthening the spleen (13). Ginseng and Atractylodes macrocephala promote gastrointestinal health (14), whereas Poria cocos helps dispel dampness and reduce edema (15). Licorice harmonizes the spleen and stomach, relieving discomfort (16). Modern pharmacological studies have shown that these herbs work synergistically to enhance intestinal motility, support digestion, maintain gut microbiota balance, and improve metabolic functions (17, 18). The active compounds in these herbs, including flavonoids, polysaccharides, saponins, and volatile oils, have demonstrated potential benefits, such as improving rumen function in ruminants and regulating metabolic processes (19–21). Based on these findings, we hypothesize that this prescription could improve postpartum NEB in dairy cows. It may achieve this by modulating the structure of the ruminal microbiota and enhancing its fermentation capacity.

Therefore, this study seeks to explore the impact of the prescription on the ruminal microbiota, fermentation capacity, and blood metabolome in postpartum dairy cows. The objective is to elucidate the mechanisms underlying its impact on postpartum NEB and to provide insights for nutritional regulation and health management of postpartum dairy cows.

## 2 Materials and methods

### 2.1 Identification of the primary active ingredients of Sijunzi San

#### 2.1.1 The formula of Sijunzi San and preparation of the solution for measurement

The Sijunzi San used in this study was prepared by Beijing Centre Biology Co. The preparation process was as follows: the herbs were washed, dried, and ground into a fine powder. Impurities were then removed using a 100-mesh sieve. Finally, in accordance with the Chinese Veterinary Pharmacopoeia, the herbs *Codonopsis pilosula*, Atractylodes macrocephala, Poria cocos, and Licorice were mixed in a ratio of 2:2:2:1 to complete the formulation.100 mg of Sijunzi San was dissolved in 1 mL of ultrapure water. The mixture was shaken for 1 min, after which 3 mL of ethanol (analytically pure) was added. The resulting solution was permitted to rest for 24 h. Subsequently, the solution was centrifuged at 3000 rpm, and the supernatant was evaporated to dryness using nitrogen, yielding a refined solution suitable for the identification of the main active ingredients.

#### 2.1.2 LC–MS/MS analysis of the refined solution of Sijunzi San

LC–MS/MS technology was utilized to identify primary active ingredients of Sijunzi San. A 0.22 μm filter membrane was used to filter the refined solution before three injections of 10 μL were made into the sample. An ultra-high-pressure liquid chromatograph (Nexera X2 LC-30, Shimadzu) was used to perform chromatographic acquisition. The ACQUITY UPLC^®^ HSS T3 chromatographic column (2.1 × 100 mm, 1.8 μm) from waters (Milford, MA, United States) was selected for this study.

Chromatographic grade formic acid in an aqueous solution of 0.1% made up mobile phase A, and chromatographic grade formic acid in an aqueous solution of 0.1% made up mobile phase B. For the first 5 min of the gradient elution, only solvent A was present in the mobile phase, with solvent B at a 0% level. From 5 to 20 min, solvent B was increased linearly from 0 to 100%. This concentration of solvent B was maintained from 20 to 25 min. Subsequently, solvent B was decreased linearly from 100% back to 0% from 25 to 25.1 min. Finally, the mobile phase was held at 0% solvent B from 25.1 to 30 min.

Samples of the refined solution were separated via UPLC and analyzed equipped with a Q Exactive Plus mass spectrometer (Thermo Scientific) with a HESI ionization source. The acquisition of the mass spectrum was performed over a duration of 30 min. The initial mass spectrometry data were refined using MSDIAL software, which helped in aligning peaks, adjusting retention times, and extracting peak areas.

### 2.2 Effects of Sijunzi San on rumen fermentation performance of dairy cows

#### 2.2.1 Collection and processing of rumen fluids in dairy cows

The experimental protocol received approval from the Animal Ethics Committee of Ningxia University (NXU-099). Rumen fluid was carefully collected from three postpartum dairy cows (600 ± 10 kg, body condition score 3.2 ~ 3.5, parity 2) through a rumen fistula, filtered through four layers of gauze, mixed in equal proportions, and promptly transferred to a preheated holding tank that was continuously purged with CO_2_ to maintain an anaerobic environment. The buffer was prepared in accordance with the method outlined by Menke et al. (22), placed in a pre-warmed, CO_2_ ventilated 1,000 mL culture flask, and subsequently mixed with rumen fluid at a 1:2 volume ratio. The resulting fermentation broth was then stored in a thermostatic water bath at 39°C.

#### 2.2.2 Test grouping

Thirty syringes were allocated to a control group (CONT) and five dosage groups for Sijunzi San (SJZS), with each group consisting of five replicates. The syringes in the SJZS groups were administered specific doses of 100, 200, 300, 400, and 500 mg, respectively, followed by the addition of 30 mL of a mixed fermentation broth to each syringe. The syringes were subsequently sealed to eliminate air and incubated in a constant-temperature shaker at 39°C for a 48 h fermentation period.

#### 2.2.3 Assessment of rumen gas production

The quantity of gas in the syringes was closely observed, and the gas yield generated by rumen fermentation was meticulously recorded at specified intervals of 0, 3, 6, 9, 12, 24, and 48 h using the scale on the syringes.

#### 2.2.4 Evaluation of rumen fermentation parameters

Following a 48 h fermentation period, pH, NH₃-N, and MCP levels were measured. A Leici PHB-4 (Shanghai, China) pH meter was used to determine the pH of rumen fluid. The NH₃-N content was assessed using the method described by Broderick and Kang (23), while the MCP content was quantified using the Thomas Brilliant Blue technique (24).

#### 2.2.5 Analysis of rumen short-chain fatty acids

Simultaneously, rumen SCFAs were quantified using a gas chromatograph (HP-INNO wax, Shimadzu Corporation, Japan). Initially, rumen fluid from each syringe was centrifuged at 5400 rpm for 10 min to eliminate impurities. Then, a 1.5 mL centrifuge tube received 1 mL of the supernatant, followed by the addition of 0.2 mL of a 25% metaphosphoric acid solution that included the internal standard, 2-ethylbutyric acid (2 EB, catalog number 80079718, Shanghai Macklin Biochemical Technology Co., Ltd., China) was added and thoroughly mixed. The sample was cooled in an ice-water bath for 30 min and centrifuged at 10,000 rpm for 10 min, after which the supernatant was extracted for analysis.

The gas chromatograph parameters were as follows: the SK-FFAP chromatographic column was used, with a column flow rate of 2.0 mL/min and an injection volume of 1 μL. The temperature program was set as follows: maintaining 120°C for 3 min, increasing to 180°C at a rate of 10°C per minute, and holding at 180°C for an additional minute. The inlet temperature was adjusted to 220°C.

For the detection instrument, the parameters were set as follows: hydrogen flow rate of 36 mL/min, air flow rate of 450 mL/min, instrument temperature of 250°C, and a column flow rate combined with a make-up gas flow rate of 40 mL/min. Standard curves for SCFAs were established using reference standards for acetate, propionate, n-butyric acid, isobutyric acid, n-valeric acid, and isovaleric acid (catalog numbers A628, P1386, B103500, 129,542, V9769, and 11,754, respectively, from Shanghai McLean Biochemical Science and Technology Co., Ltd., China). The peak area of these standards served as the vertical coordinates, while their concentrations were plotted as the horizontal coordinates, enabling the derivation of a linear regression equation for SCFAs (Supplementary Table S1). The concentrations of SCFAs in the rumen fluids were subsequently calculated using this standard curve.

### 2.3 The impact of Sijunzi San on the composition and abundance of rumen microflora

Following a 48 h fermentation period, the ideal dosage of Sijunzi San was determined drawing from the outcomes of rumen fermentation parameters and SCFA levels. Rumen fluids containing this optimal dosage of Sijunzi San were selected to assess the prescription’s influence on the structure and abundance of the ruminal bacterial community using 16S rRNA high-throughput sequencing technology.

Initially, DNA was extracted from the rumen fluid employing the magnetic bead method. The procured DNA was subsequently measured with a Nanodrop instrument, and its wholeness was assessed via 1.2% agarose gel electrophoresis. The DNA was subsequently diluted to a concentration of 20 ng/μL. Standard bacterial primers (upstream: ACTCCTACGGGGAGGCAGCA; downstream: GGACTACHVGGGTWTCTAAT) and an amplification polymerase were used to amplify the V3–V4 region of bacterial 16S rRNA (25). The PCR-amplified products were quantified through fluorescence. The volume of sequencing required for each rumen fluid specimen was set according to the data obtained from fluorescence measurements. The samples were then mixed in their designated proportions. Sequencing libraries for each rumen fluid sample were constructed employing Illumina’s TruSeq Nano DNA LT Library Prep Kit, which streamlined the process for high-throughput sequencing.

The QIIME2 (2019.4) software was used to initially conduct species taxonomic annotation on rumen microbial 16S rRNA gene sequences, followed by analyses of Alpha and Beta diversity (26, 27). The Spearman’s correlation heatmaps, illustrating the interrelationships between the primary active ingredients and the ruminal differential microbes, as well as between these microbes and ruminal SCFAs, were generated using the OmicShare Tools.^1^

### 2.4 The impacts of Sijunzi San on immune function, lipid metabolism, and blood metabolome of postpartum dairy cows

#### 2.4.1 Grouping of experimental animals and collection of blood samples

At a large-scale intensive dairy farm in Ningxia, China, 16 robust postpartum Holstein cows, each weighing 600 ± 30 kg with body condition scores ranging from 3.0 to 3.5 and having 2 to 3 lactations, were randomly assigned to either a control group (CONT) or a Sijunzi San group (SJZS), with equal representation in each group. Both groups were fed the same total mixed ration (TMR) and had ad libitum access to water. The dosage of 300 g per day was determined based on the medication guidelines recommended by the Chinese Veterinary Pharmacopoeia. In the SJZS group, cows were administered 300 g of Sijunzi San mixed with 5 L of water via a gastric tube before their morning feeding, for a period of 7 days. In contrast, cows in the CONT group were provided with an equivalent quantity of plain drinking water. Prior to morning feeding on the eighth day, blood samples were collected from the tail vein of each cow. Plasma preparation involved centrifugation at 3,000 rpm for 20 min at a chilled 4°C, after which it was kept at −80°C for impending examination of biochemical indices and metabolite levels.

#### 2.4.2 Evaluation of immunological parameters

The concentrations of IgA, IgG, and IgM in the plasma of cows were quantified using ELISA kits (product numbers YJ542063, YJ330698, and YJ627279), acquired from Shanghai Enzyme-Link Biotechnology Co., Ltd., an enterprise based in China.

#### 2.4.3 Inspection of lipid metabolic indicators

Blood KET levels were measured using a blood ketone meter (AHM001P, Colibri, Wuhan, China). ELISA kits were utilized to assess blood concentrations of NEFA (A042-2-1, Nanjing Jiancheng Bioengineering Institute, China) and MTTP (NWLT, Shanghai Enzyme-linked Biotechnology Co., Ltd., China). Additionally, biochemical kits were employed to measure TG levels in the blood (A110-1-1, Nanjing Jiancheng Bioengineering Institute, China).

#### 2.4.4 Blood metabolomics analysis

UHPLC–MS/MS was employed to conduct a non-targeted blood metabolomic analysis. A 100 μL aliquot of plasma was added to a 1.5 mL polyethylene (PE) tube containing 400 μL of pre-cooled, highly concentrated methanol (1.06007.4008, Millipore, United States) and acetonitrile (1.00030.4008, Millipore, United States) mixed in a 1:1 (v/v) ratio. The mixture underwent a 1 h cooling phase in an ice-water bath before being centrifuged at 14,000 g at 4°C for 20 min, leading to the isolation of the supernatant for subsequent analysis.

Chromatographic data acquisition was performed using an ACQUITY UPLC HSS T3 column (2.1 × 100 mm, 1.8 μm) from waters, with a flow rate of 0.3 mL/min. The mobile phases consisted of 0.1% chromatographic-grade formic acid (A) and 100% chromatographic-grade acetonitrile (B). The elution gradient was initiated at 0% B for 2 min, followed by a linear increase to 48% B over the next 4 min. Subsequently, the concentration of B was gradually increased to 100% within an additional 2 min. Following this, the concentration of B was reduced to 0% over a period of 0.1 min, and then equilibrated for 3 min. In the MS analysis, ESI was applied across positive and negative modes, with the HESI source parameters detailed below: spray voltages of 3.8 kV and 3.2 kV for positive and negative modes; a capillary temperature of 320°C; sheath and auxiliary gas flows, nitrogen, at 30 arb; probe heater temperature at 350°C; and S-Lens RF level adjusted to 50.

The UHPLC–MS/MS data acquired were processed using R (version 4.0.3) along with its affiliated software packages to perform Principal Component Analysis (PCA) and Orthogonal Partial Least Squares Discriminant Analysis (OPLS-DA) (28). PCA was employed to analyze the variability between and within groups. OPLS-DA was utilized to construct a discriminant model based on the Variable Importance for the Projection (VIP) values of blood metabolites. Furthermore, the Fold Change (FC) values and *p*-values derived from univariate analysis were integrated to identify differential metabolites. Subsequently, KEGG pathway enrichment analysis of these metabolites was conducted using MetaboAnalyst^2^ (29).

### 2.5 Statistical analysis

All data were analyzed using GraphPad Prism version 8.0.2 (GraphPad Software). All experimental data are presented as the mean ± standard deviation (SD). one-way ANOVA was used to compare the data between the six groups, and independent samples t-test was used to analyze the data between the two groups. Differential analysis of species abundance in rumen microbiota was performed using the Wilcoxon Mann–Whitney test. *p* < 0.05 was considered statistically significant.

## 3 Results

### 3.1 Main active ingredients of Sijunzi San

**Figure 1:**
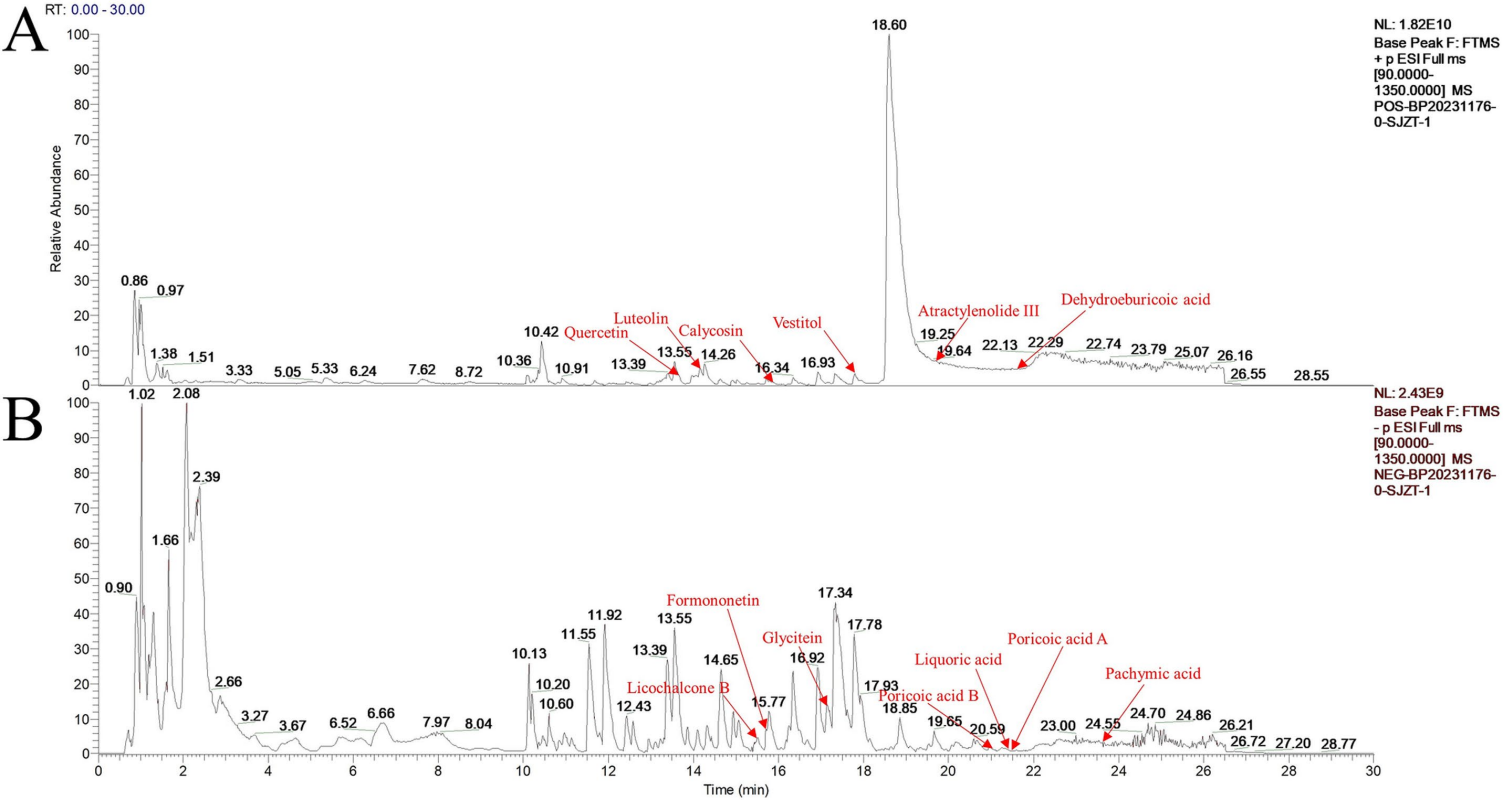
Base peak chromatograms of positive and negative ion modes of Sijunzi San. **(A)** Positive ion mode; **(B)** Negative ion mode.

Thirteen primary active ingredients of Sijunzi San were identified using LC–MS/MS, including luteolin, glycitein, pachymic acid, poricoic acid A, poricoic acid B, dehydroeburicoic acid, atractylenolide III, formononetin, calycosin, licochalcone B, liquoric acid, quercetin, and vestitol (Figures 1A,B). The retention time (RT), molecular weight (m/z), ion mode, formula, class, and origin of the main active ingredients are detailed in Table 1.

**Table 1:** Information of main active ingredients of Sijunzi San.

Number	Identification	RT	m/s	Ion mode	Formula	Class	Origin
1	Luteolin	13.995	287.05499	[M+H]+	C15H10O6	Flavonoids	*Codonopsis pilosula*
2	Glycitein	17.197	283.06033	[M−H]−	C16H12O5	Isoflavonoids	*Codonopsis pilosula*
3	Pachymic acid	23.596	527.37488	[M−H]−	C33H52O5	Prenol lipids	Atractylodes macrocephala; *Codonopsis pilosula*
4	Poricoic acid A	21.485	497.32761	[M−H]−	C31H46O5	Prenol lipids	Atractylodes macrocephala; *Codonopsis pilosula*
5	Poricoic acid B	21.058	483.31198	[M−H]−	C30H44O5	Prenol lipids	Atractylodes macrocephala; *Codonopsis pilosula*
6	Dehydroeburicoic acid	21.651	469.36707	[M+H]+	C31H48O3	Steroids and steroid derivatives	Atractylodes macrocephala; *Codonopsis pilosula*
7	Atractylenolide III	19.646	249.14865	[M+H]+	C15H20O3	Prenol lipids	*Codonopsis pilosula*; Poria cocos
8	Formononetin	15.721	267.06552	[M−H]−	C16H12O4	Isoflavonoids	Licorice; *Codonopsis pilosula*
9	Calycosin	16.028	285.07513	[M+H]+	C16H12O5	Isoflavonoids	Licorice; *Codonopsis pilosula*
10	Licochalcone B	15.527	285.07651	[M−H]−	C16H14O5	Linear 1,3-diarylpropanoids	Licorice
11	Liquoric acid	21.433	483.31207	[2 M−H]−	C30H44O5	Prenol lipids	Licorice
12	Quercetin	13.639	303.04922	[M+H]+	C15H10O7	Flavonoids	Licorice; Poria cocos; *Codonopsis pilosula*
13	Vestitol	17.861	273.11133	[M+H]+	C16H16O4	Isoflavonoids	Licorice

### 3.2 The impact of Sijunzi San on rumen fermentation in dairy cows

#### 3.2.1 The effect of Sijunzi San on rumen gas production

**Figure 2:**
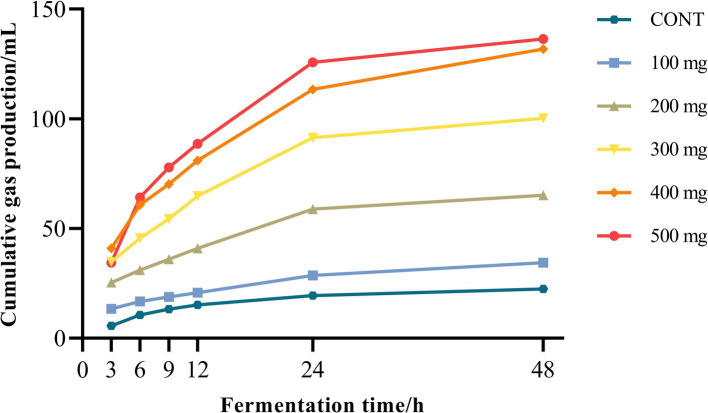
Effect of different doses of Sijunzi San on rumen gas production in dairy cows (*n* = 5). CONT represented control group and 100–500 mg represented different doses groups of Sijunzi San.

Rumen gas production in both the control group and the groups treated with varying doses of Sijunzi San progressively increased with the extension of fermentation time over a 48 h period, showing a sharp rise from 0 to 12 h, followed by a plateau from 12 to 48 h. At corresponding fermentation time points, rumen gas generation was significantly enhanced with the administration of varying dosages of Sijunzi San as opposed to the control group (*p* < 0.05). Moreover, within the dose range of 200 to 500 mg, Sijunzi San demonstrated a notable dose-dependent effect at the fermentation time points of 3, 6, 9, 12, 24, and 48 h (Figure 2).

#### 3.2.2 The impact of Sijunzi San on rumen fermentation parameters

**Figure 3:**
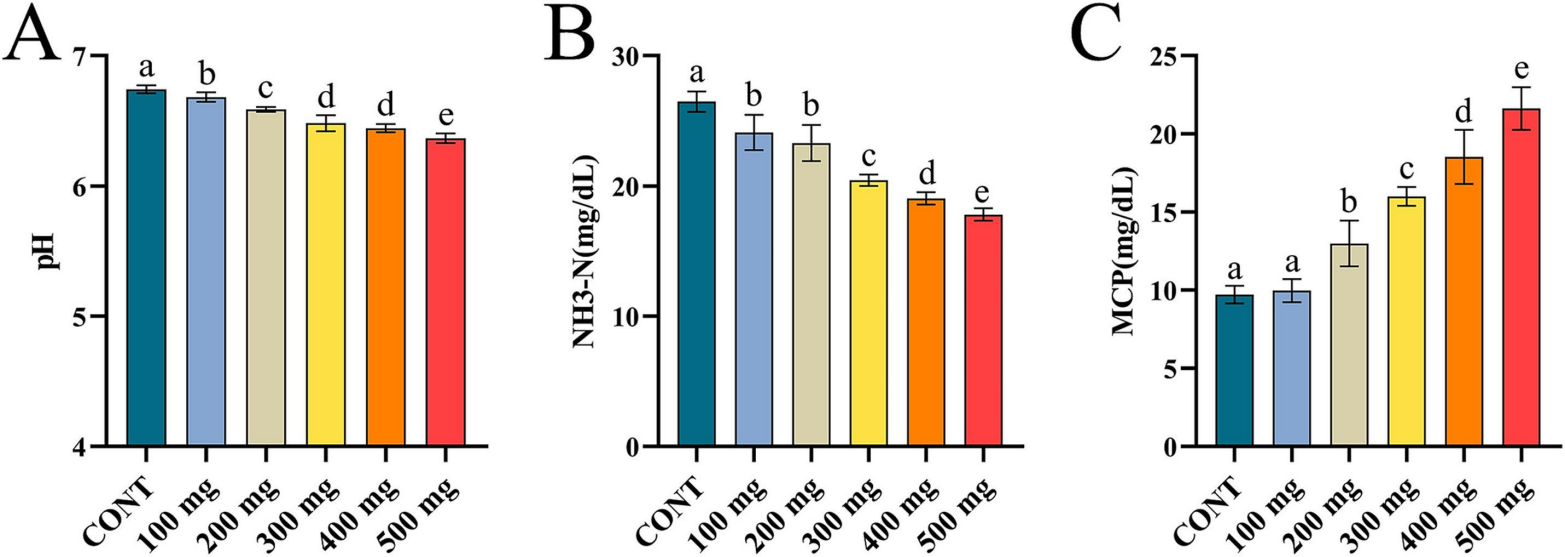
Effect of Sijunzi San on rumen fermentation parameters in dairy cows (*n* = 5). CONT represented control group, 100 ~ 500 mg represented different doses of Sijunzi San; **(A)** pH; **(B)** NH_3_-N; **(C)** MCP, microbial protein. Different letters indicated significant differences (*p* < 0.05), while the same letters indicated non-significant differences (*p* > 0.05).

The pH and NH₃-N levels in the rumen were significantly reduced (*p* < 0.05), while MCP contents were notably elevated (*p* < 0.05) in the groups treated with varying doses of Sijunzi San compared to the control group. As the medicinal amount of Sijunzi San augmented, there was a significant lessen in pH and NH₃-N levels and a significant rise in MCP content (*p* < 0.05). The values of pH, NH₃-N, and MCP all demonstrated a strong dose-dependent correlation (Figures 3A–C). This indicates that Sijunzi San can markedly enhance rumen fermentation capacity in dairy cows, with the most pronounced effect observed at a dose of 500 mg.

#### 3.2.3 The influence of Sijunzi San on rumen SCFAs

**Figure 4:**
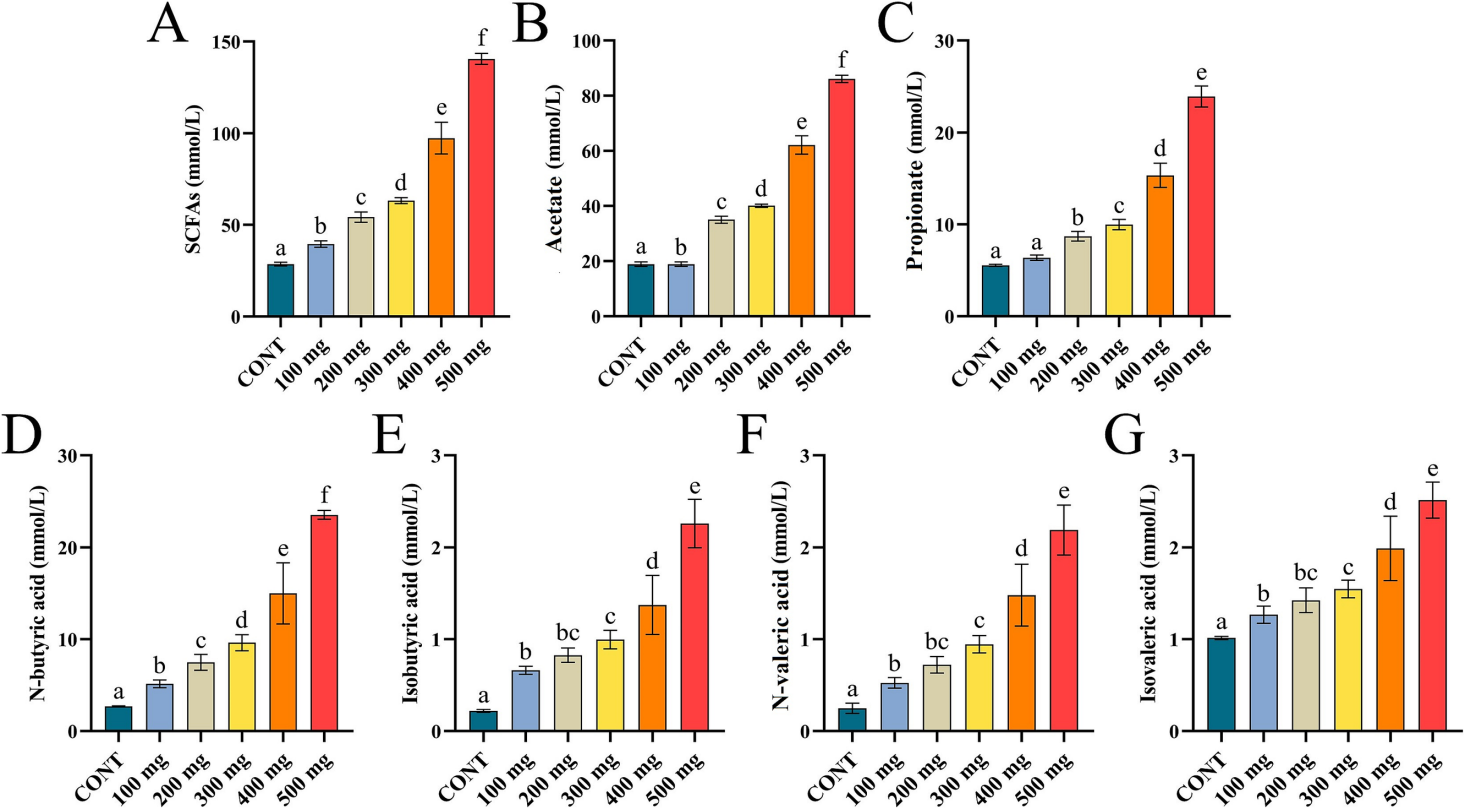
Effect of Sijunzi San on SCFAs in the rumen of dairy cows (*n* = 5). CONT represented control group, 100 ~ 500 mg represented different doses of Sijunzi San; **(A)** SCFAs; **(B)** Acetate; **(C)** Propionate; **(D)** N-butyric acid; **(E)** Isobutyric acid; **(F)** N-valeric acid; **(G)** Isovaleric acid. Different letters indicated significant differences (*p* < 0.05), while the same letters indicated non-significant differences (*p* > 0.05).

Compared to the control group, the contents of ruminal SCFAs, acetate, propionate, and n-butyric acid in the groups treated with various dosages of Sijunzi San were significantly higher (*p* < 0.05), demonstrating a robust dose-dependent response. In different doses of Sijunzi San, the concentrations of isobutyric acid, n-valeric acid, and isovaleric acid gradually increased, and a good dose-dependent relationship was observed within the range of 300 mg to 500 mg. These results indicated that Sijunzi San effectively promotes the production of rumen SCFAs, with the most significant effect observed at a dose of 500 mg (Figures 4A–G).

### 3.3 Effect of Sijunzi San on the composition and abundance of rumen microbiota in dairy cows

#### 3.3.1 Ruminal microbiota diversity

**Figure 5:**
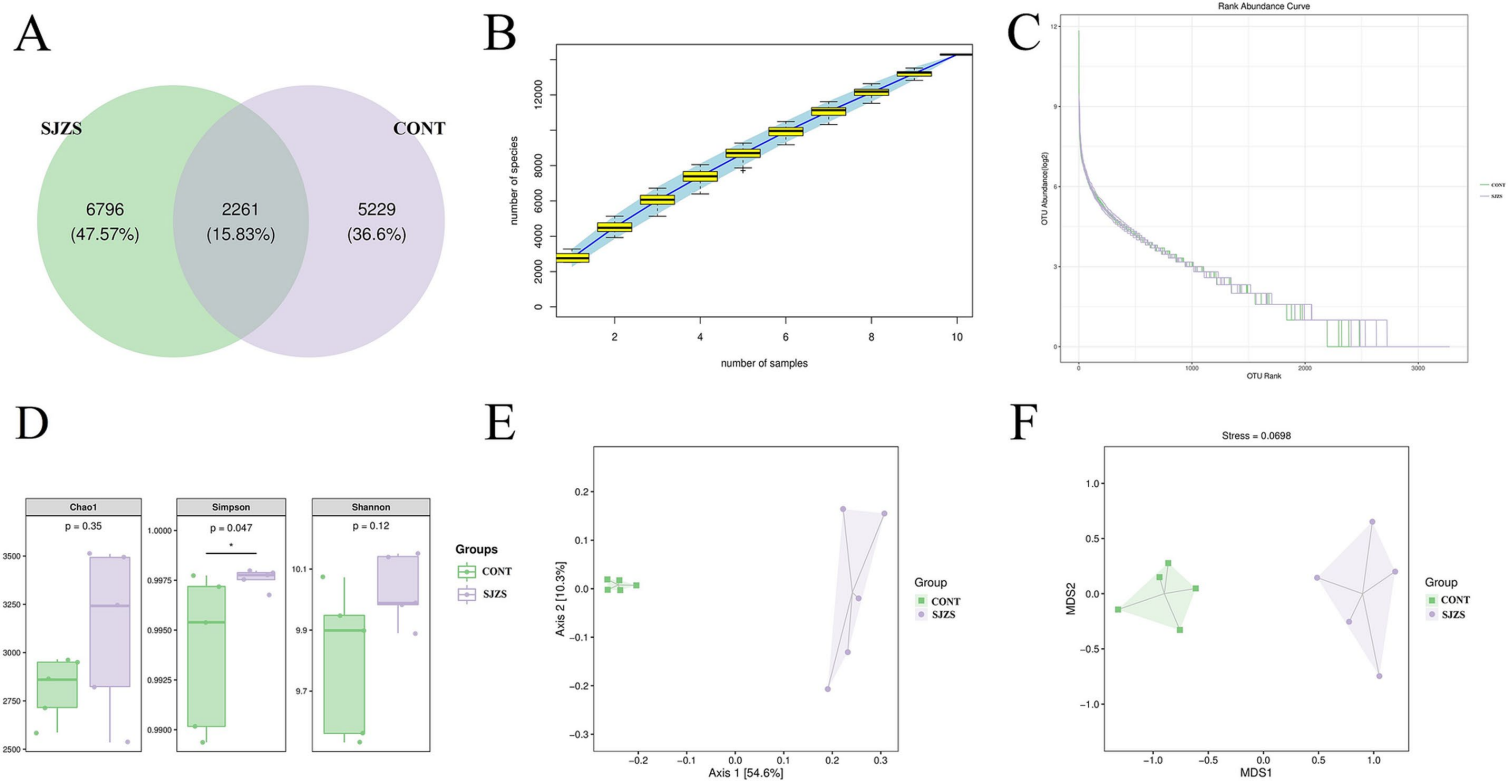
Effect of Sijunzi San on rumen microbiota diversity of dairy cows. CONT represented the control group and SJZS represented the Sijunzi San group. **(A)** OTU Venn plots of rumen microorganisms in two groups. **(B)** Specaccum species cumulative plots, with the sample size as the horizontal coordinate and the number of observed species (ASV/OTU) as the vertical coordinate. The blue shading reflects the confidence intervals for the curves. **(C)** Abundance Ranking plot, with the horizontal coordinate being the ordinal number of ASVs/OTUs in order of abundance and the vertical coordinate being the log2 value of the abundance of each ASV/OTU in the group. **(D)** Alpha diversity analysis. The Chao1 index is used to assess the total number of OTUs present in the community. The Simpson index is used to assess community evenness. And the Shannon index is used to combine the assessment of the community’s richness and evenness. **(E)** Principal Coordinate Analysis (PCoA) of two groups of rumen microbes. **(F)** Non-Metric Multidimensional Scale Analysis (NMDS) of two groups of rumen microbes.

This study selected samples from the 500 mg dosage group based on the previous results to explore the impact of Sijunzi San on the compound and quantity of rumen microbiota. A total of 14,286 Operational Taxonomic Units (OTUs) were identified from the rumen fluids of two groups. Among these, the Sijunzi San group contained 9,057 OTUs, while the control group contained 7,490 OTUs. A total of 2,261 OTUs were shared between the two groups, constituting 15.83% of the overall OTUs (Figure 5A). The species accumulation curve approached a plateau as the sample size increased (Figure 5B), and the abundance grade curve declined gently, indicating high sample diversity (Figure 5C). This indicates that the sequencing coverage was adequate to precisely depict the compositional structure of the rumen microbial community. Alpha diversity analysis demonstrated that the Simpson’s index for the Sijunzi San group was obviously rised compared to the control group (*p* < 0.05) (Figure 5D), suggesting that Sijunzi San effectively enhanced the abundance and diversity of the rumen microbiota. The Principal Coordinate Analysis (PCoA) and Non-Metric Multidimensional Scale (NMDS) Analysis visualizations derived from the Beta diversity analysis clearly demonstrated a clear separation in the constitution and abundance of rumen microbiota among the two groups (Figures 5E,F), pointing to a substantial impact of Sijunzi San on the composition and quantity of rumen microbiota.

#### 3.3.2 Differences in rumen microbiota

**Figure 6:**
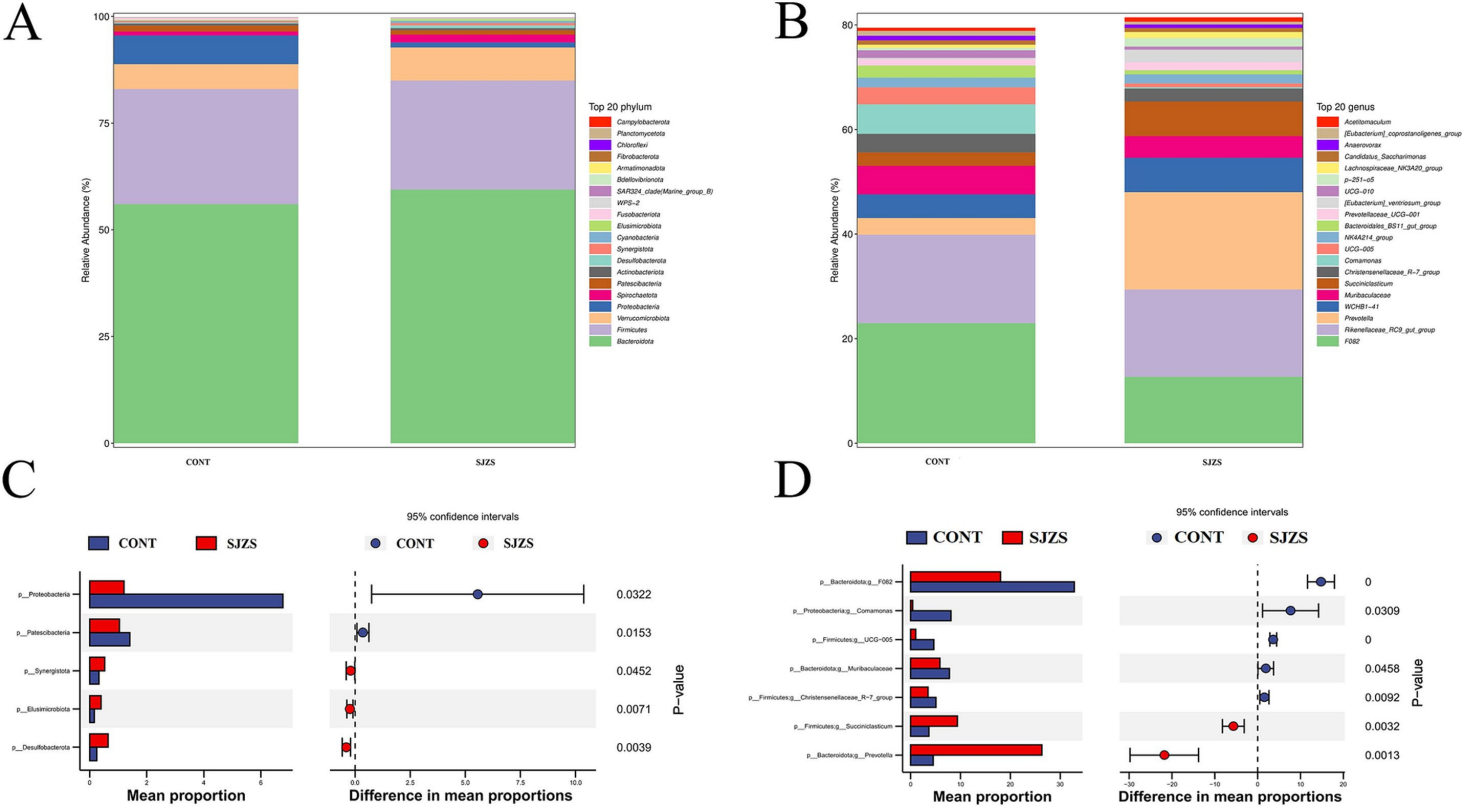
Differential microbiota analysis of Sijunzi San on rumen microbiota composition in postpartum dairy cows. CONT represented the control group and SJZS represented the Sijunzi San group. **(A,B)** Histograms of species composition abundance for the TOP20 at phylum level and genus level respectively, with each horizontal bar representing a species. **(C,D)** Graphs of Stamp species difference analysis based on phylum level and genus level, respectively.

The predominant phyla in both groups were Bacteroidota and Firmicutes, accounting for 56.0 and 26.9% of the microbiota in the control group, and 59.4 and 25.6% in the Sijunzi San group, respectively (Figure 6A). The rumen microbiota in both groups were predominantly consisted of *F082*, *Rikenellaceae_RC9_gut_group* and *Prevotella* genus. In the control group, these genera constituted 22.9, 16.9, and 3.18% of the microbiota, respectively. In the Sijunzi San group, their proportions were 12.7, 16.6, and 18.6%, respectively (Figure 6B). The results of Stamp species difference analysis revealed that, compared to the control group, the Sijunzi San group exhibited significantly higher relative abundances of the Synergistota, Elusimicrobiota, and Desulfobacterota phyla (*p* < 0.05). In contrast, the relative abundances of the Proteobacteria and Patescibacteria phyla were notably reduced in the Sijunzi San group (*p* < 0.05) (Figure 6C). Additionally, compared to the control group, the relative abundances of *Succiniclasticum* (6.6%) and *Prevotella* (18.6%) genera within the Sijunzi San group were significantly elevated (*p* < 0.05). Conversely, the relative abundances of the *Muribaculaceae*, *UCG-005*, *Christensenellaceae_R-7_group*, *Comamonas*, and *F082* genera were notably diminished (*p* < 0.05) (Figure 6D).

#### 3.3.3 Correlation analysis of differential microorganisms with rumen SCFAs and active ingredients of Sijunzi San

**Figure 7:**
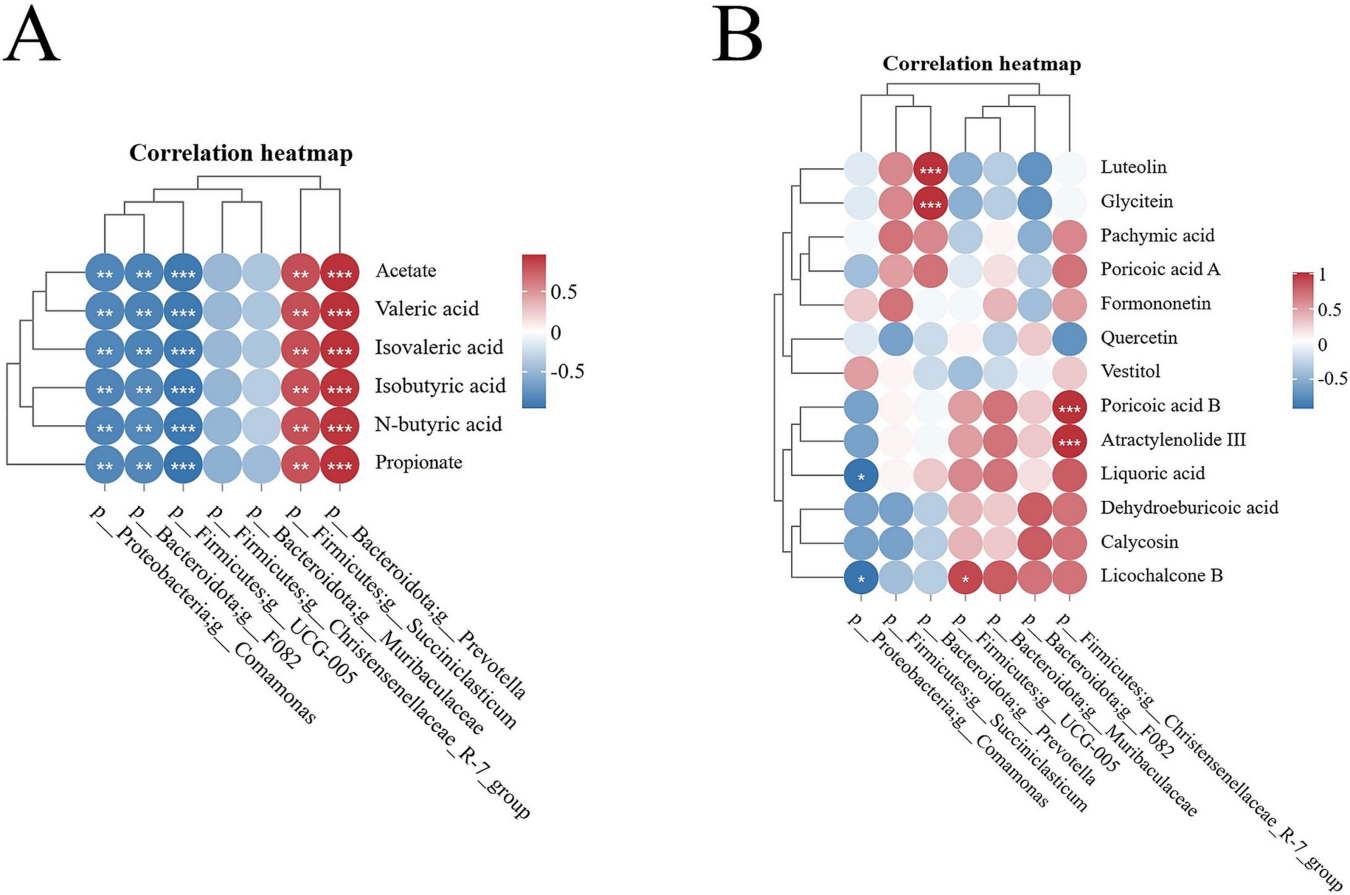
Spearman correlation heatmap. **(A)** Heatmap of correlation between ruminal differential microbes and SCFAs; **(B)** Heatmap of correlation between active ingredients in Sijunzi San and ruminal differential microbes. Red indicated positive correlation, blue indicated negative correlation, * indicated *p* < 0.05, ** indicated *p* < 0.01 and *** indicated *p* < 0.001.

The heatmap of the Spearman correlation coefficients displayed that *Succiniclasticum* and *Prevotella* genera exhibited a significant positive correlation with rumen SCFAs (*p* < 0.05). Conversely, the genera *UCG-005*, *Comamonas*, and *F082* demonstrated a significant negative correlation with rumen SCFAs (*p* < 0.05). The genera *Muribaculaceae* and *Christensenellaceae_R-7_group* did not show a significant correlation with rumen SCFAs (*p* > 0.05) (Figure 7A; Supplementary Table S2).

The active ingredients luteolin and glycitein of Sijunzi San in Sijunzi San exhibited a significant positive correlation the genus *Prevotella* (*p* < 0.05). Similarly, poricoic acid B and Atractylenolide III demonstrated a significant positive correlation with the genus *Christensenellaceae_R-7_group* (*p* < 0.05). Additionally, licochalcone B was found to have a significant positive correlation with the genus *UCG-005* (*p* < 0.05). Conversely, liquoric acid and licochalcone B were significantly negatively correlated with the genus *Comamonas* (*p* < 0.05) (Figure 7B; Supplementary Table S3).

### 3.4 Effect of Sijunzi San on immunity, lipid metabolism, and blood metabolome in postpartum dairy cows

#### 3.4.1 The impacts of Sijunzi San on blood IgA, IgG, and IgM

**Figure 8:**
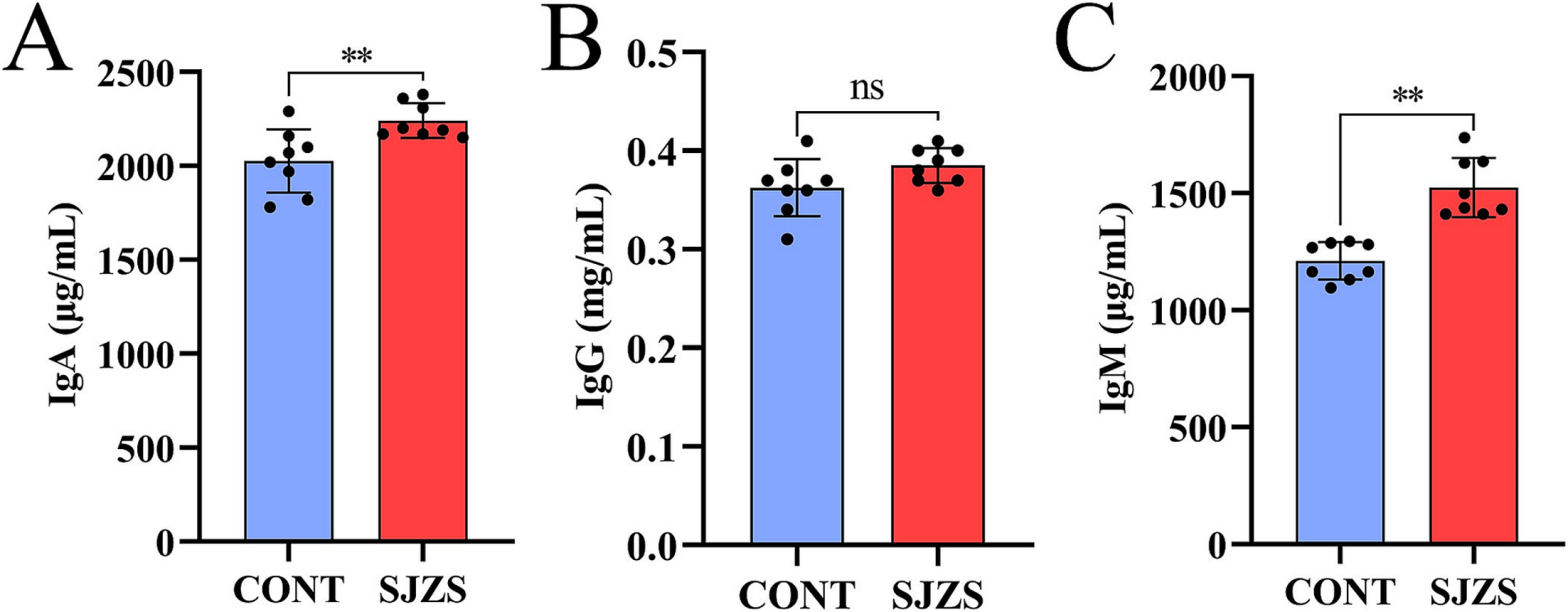
Effect of Sijunzi San on immune response of postpartum dairy cows (*n* = 8). CONT represented control group, SJZS represented Sijunzi San group; **(A)** IgA; **(B)** IgG; **(C)** IgM. Each black dot represented a sample, * indicated *p* < 0.05 and ** indicated *p* < 0.01.

Compared with the control group, the postpartum cows in the Sijunzi San group exhibited elevated levels of IgA, IgG, and IgM in their blood. Notably, the increases in IgA and IgM were highly significant (*p* < 0.01), as illustrated in Figures 8A–C. This suggested that Sijunzi San enhanced the immune response of postpartum dairy cows to some extent.

#### 3.4.2 Effects of Sijunzi San on blood KET, NEFA, TG, and MTTP in postpartum dairy cows

**Figure 9:**
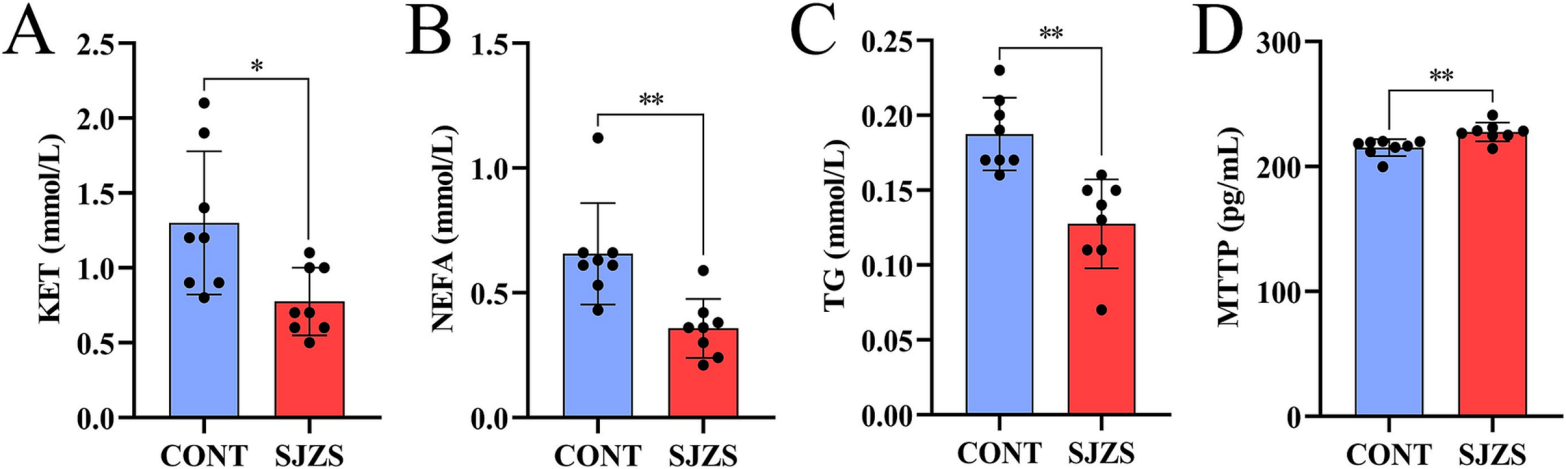
Effect of Sijunzi San on blood lipid metabolism of postpartum dairy cows (*n* = 8). CONT represented control group, SJZS represented Sijunzi San group; **(A)** KET: ketones; **(B)** NEFA: non-esterified fatty acids; **(C)** TG: triglycerides; **(D)** MTTP: microsomal triglyceride transfer protein; Each black dot represented one sample, * indicated *p* < 0.05, ** indicated *p* < 0.01.

Compared with the control group, the postpartum cows in the Sijunzi San group showed a significant reduction in KET levels (*p* < 0.05), an extremely significant decrease in NEFA and TG concentrations (*p* < 0.01), and an extremely significant elevation in MTTP content (*p* < 0.01) as depicted in Figures 9A–D. These findings suggested that Sijunzi San exerts a definite ameliorative effect on lipid mobilization in postpartum cows.

#### 3.4.3 The impact of Sijunzi San on the blood metabolomic profile of postpartum dairy cows

##### 3.4.3.1 Metabolic profile alterations and differential metabolite identification

Non-targeted metabolomics with UHPLC–MS/MS was employed to analyze plasma samples, observing the impact of Sijunzi San on the blood metabolic profile of postpartum dairy cows, revealing a total of 283 blood metabolites (Supplementary Figures S1A–C).

**Figure 10:**
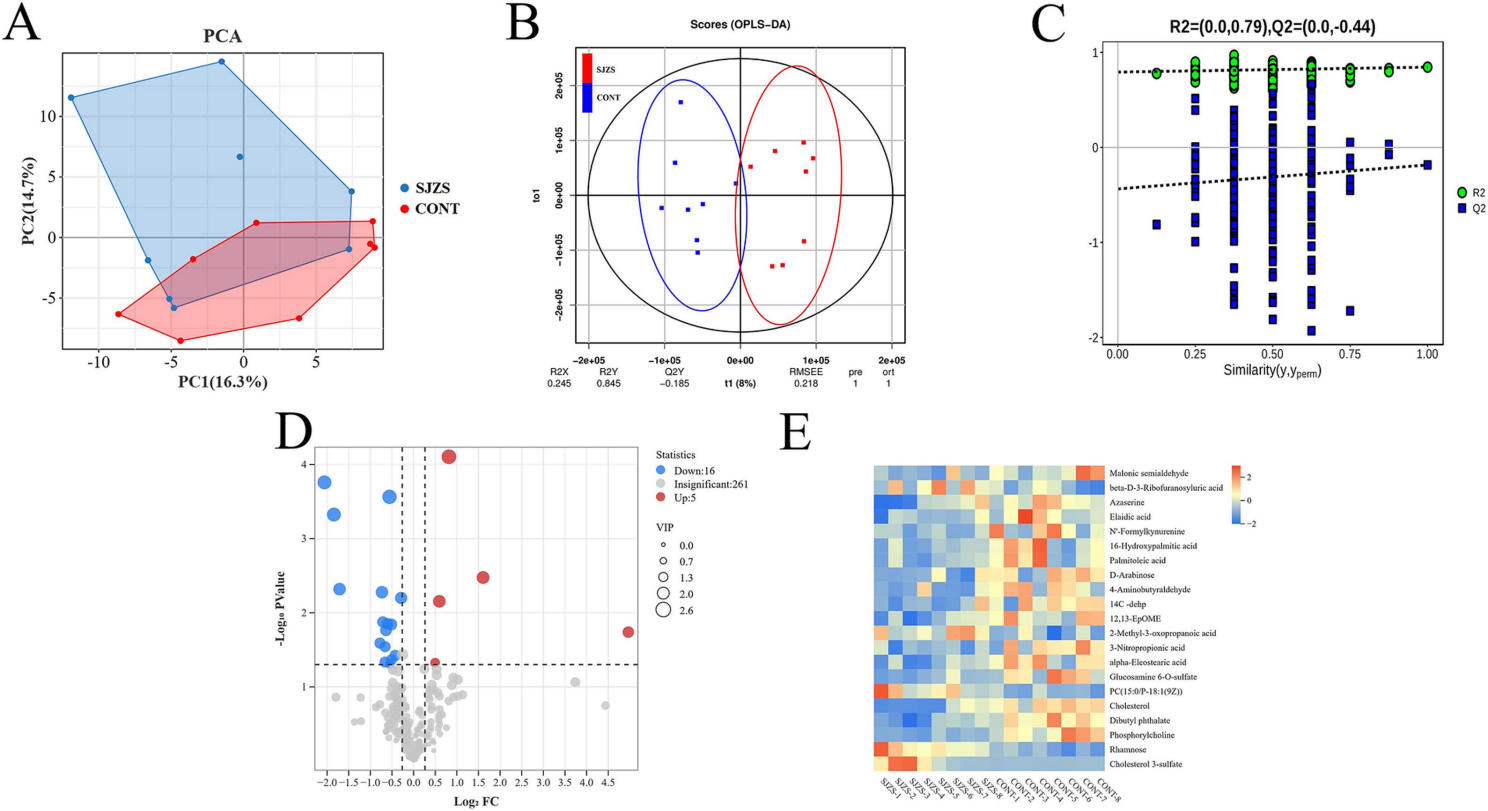
Effect of Sijunzi San on blood metabolome of postpartum dairy cows. CONT represented the control group and SJZS represented the Sijunzi San group. **(A)** Principal Component Analysis (PCA) demonstrating the trend of intra-group aggregation and inter-group separation of the two groups of rumen fluid samples. **(B)** Orthogonal Partial Least Squares Discriminant Analysis (OPLS-DA) score plot demonstrating the trend of separation of the two groups of rumen fluid samples. **(C)** OPLS-DA permutation test plot for seeing whether overfitting occurred in the OPLS-DA model. **(D)** Volcano plot demonstrating the trend of differential metabolites, with each dot representing a metabolite. Red indicated up-regulation and blue indicated down-regulation, and the size of the dots indicated the Variable importance for the projection (VIP) value. **(E)** Clustering heatmap of differential metabolites, with the horizontal coordinate indicating the samples and the vertical coordinate indicating the metabolites. Red cooler indicated up-regulation and blue cooler indicated down-regulation.

The PCA plot indicated a discernible trend of differentiation between the Sijunzi San group and the control group, with PC1 accounting for 16.3% and PC2 for 14.7% of the variance (Figure 10A). The OPLS-DA model, constructed to scrutinize the importance of metabolites in classification (Figure 10B), was confirmed to be valid and free from overfitting, as evidenced by the permutation test diagram where the intercept between Q2 and the Y-axis was less than zero (R2 = 0.79, Q2 = −0.44) (Figure 10C). Metabolites with VIP more than 1 (VIP ≥ 1) were chosen from the OPLS-DA model for subsequent univariate analysis, to ascertain the FC and *p* value for each metabolite (Figure 10D). A total of 21 differential metabolites were identified with VIP more than 1 (VIP ≥ 1), *p* value less than 0.05 (*p* < 0.05) and FC less than 0.83 (FC ≤ 0.83) or FC more than 1.2 (FC ≥ 1.2). Among these, 5 metabolites exhibited significant up-regulation, while 16 showed significant down-regulation. The Rt (min), Mean, VIP, FC, *p*-value, and False Discovery Rate (FDR) of differential metabolites were shown in Table 2. The clustering heatmap showed that the 21 differential metabolites identified as differentially expressed exhibited excellent within-group consistency and were distinctly separated between the two groups (Figure 10E), indicating that the selected metabolites were indeed representative.

**Table 2:** Blood differential metabolites and related parameters.

Metabolites	Rt (min)	Average peak area		VIP^1^	FC^2^	*p*-value	FDR
		SJZS	CONT				
Cholesterol 3-sulfate	13.464	4.91E+08	1.57E+07	1.873	31.223 ↑	0.018	1.873
PC(15:0/P-18:1(9Z))	12.429	1.63E+07	5.35E+06	2.195	3.044↑	0.003	2.195
Rhamnose	1.176	1.35E+08	7.66E+07	2.634	1.762↑	0.000	2.634
2-Methyl-3-oxopropanoic acid	8.495	4.23E+07	2.80E+07	2.146	1.510↑	0.007	2.146
beta-D-3-Ribofuranosyluric acid	8.115	3.53E+07	2.50E+07	1.449	1.412↑	0.047	1.449
3-Nitropropionic acid	13.499	5.03E+08	6.14E+08	2.060	0.819↓	0.006	2.060
N′-Formylkynurenine	8.178	4.80E+07	6.45E+07	1.845	0.745↓	0.037	1.845
Elaidic acid	12.621	1.17E+08	1.67E+08	1.710	0.701↓	0.043	1.710
4-Aminobutyraldehyde	14.478	2.52E+07	3.61E+07	1.976	0.699↓	0.014	1.976
Malonic semialdehyde	1.337	7.96E+07	1.16E+08	1.415	0.683↓	0.049	1.415
Dibutyl phthalate	12.523	2.11E+09	3.11E+09	2.578	0.680↓	0.000	2.578
14C-dehp	12.596	2.96E+07	4.51E+07	1.934	0.657↓	0.014	1.934
D-Arabinose	1.016	3.97E+07	6.18E+07	1.927	0.643↓	0.017	1.927
16-Hydroxypalmitic acid	12.203	9.85E+07	1.56E+08	1.786	0.633↓	0.029	1.786
Azaserine	8.826	1.89E+07	2.98E+07	1.757	0.632↓	0.046	1.757
12,13-EpOME	12.468	3.08E+07	5.05E+07	1.849	0.611↓	0.013	1.849
Alpha-Eleostearic acid	12.425	3.83E+07	6.35E+07	2.114	0.602↓	0.005	2.114
Palmitoleic acid	12.204	2.15E+07	3.68E+07	1.811	0.584↓	0.026	1.811
Glucosamine 6-O-sulfate	8.915	9.60E+06	3.14E+07	2.136	0.305↓	0.004	2.136
Cholesterol	13.301	1.27E+08	4.56E+08	2.471	0.279↓	0.001	2.471
Phosphorylcholine	12.249	6.10E+07	2.55E+08	2.465	0.240↓	0.000	2.465

(1) The variable importance for the projection (VIP) of differential metabolites calculated based on orthogonal partial least squares discriminant analysis (OPLS-DA). (2) Fold change of differential metabolites calculated based on relative peak intensity. ↑ Indicated significant up-regulation and ↓ indicated significant down-regulation.

##### 3.4.3.2 Pathway enrichment analysis

**Figure 11:**
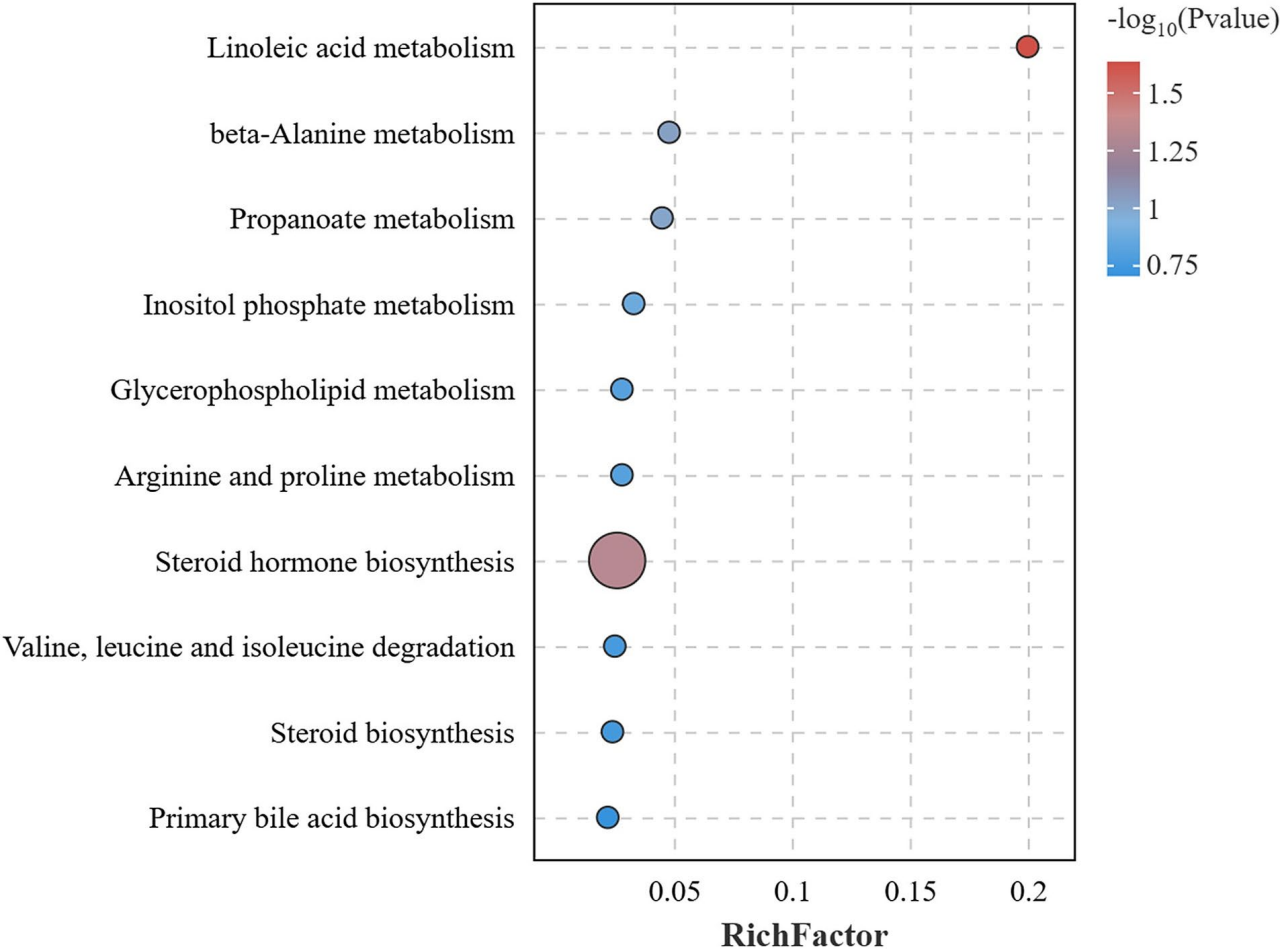
KEGG pathway enrichment analysis of differential metabolites. Bubble plot of KEGG enrichment analysis, the size of the dots indicated the number of enriched differential metabolites, and the color shade of the dots indicated the significance of the pathway.

KEGG pathway enrichment was performed for 21 different metabolites. The analysis uncovered that seven of these metabolites, namely 12(13)-EpOME, Cholesterol, Cholesterol 3-sulfate, Malonic semialdehyde, Phosphorylcholine, 4-Aminobutyraldehyde, and 2-Methyl-3-oxopropanoic acid, were significantly enriched, suggesting a notable impact on linoleic acid metabolism and steroid hormone biosynthesis within lipid metabolic pathways (*p* < 0.05) (Table 3). Additionally, these metabolites appear to potentially influence on other lipid metabolic pathways, such as glycerophospholipid metabolism, steroid biosynthesis, and primary bile acid biosynthesis. They also potentially impact some amino acid metabolic pathways, such as beta-alanine metabolism, propanoate metabolism, inositol phosphate metabolism, arginine and proline metabolism, as well as the degradation pathways of valine, leucine, and isoleucine (Figure 11).

**Table 3:** Metabolic pathways and parameters related to differential metabolites.

Metabolic pathway	*p*-value	Impact	Relevant metabolites
Linoleic acid metabolism	0.022	0.000	12(13)-EpOME
Steroid hormone biosynthesis	0.045	0.006	Cholesterol; Cholesterol 3-sulfate
beta-Alanine metabolism	0.092	0.104	Malonic semialdehyde
Propanoate metabolism	0.096	0.005	Malonic semialdehyde
Inositol phosphate metabolism	0.129	0.000	Malonic semialdehyde
Glycerophospholipid metabolism	0.153	0.009	Phosphorylcholine
Arginine and proline metabolism	0.153	0.066	4-Aminobutyraldehyde
Valine, leucine and isoleucine degradation	0.169	0.009	2-Methyl-3-oxopropanoic acid
Steroid biosynthesis	0.173	0.028	2-Methyl-3-oxopropanoic acid
Primary bile acid biosynthesis	0.192	0.033	2-Methyl-3-oxopropanoic acid

## 4 Discussion

### 4.1 Impact of Sijunzi San on ruminal fermentation performance of dairy cows

Gas production, a comprehensive indicator of the degree of rumen fermentation in ruminants, is positively correlated with rumen microbiota activity. Increased gas production reflects a higher content of fermentable carbohydrates in the feed and a boost in the activity of the rumen microbiota (30, 31). Antonius et al. (32) reported that herbs such as Moringa, Hairy White Redwood, and Turmeric significantly increased gas production *in vitro* rumen fermentation. Peng et al. (33) discovered that the addition of Astragalus to an *in vitro* rumen trial substantially boosted total gas production after 6 h of fermentation. The study found that after the addition of the Sijunzi San, the gas production from rumen fermentation gradually increased, then rose rapidly, ultimately stabilizing within 48 h, showing a distinct dose-dependent response. This suggests that Sijunzi San has the potential to enhance rumen substrate fermentation.

Ruminal pH, NH₃-N, and MCP are also important indicators of rumen microbial fermentation. Ruminal pH is essential for maintaining normal physiological functions in ruminants, with stable pH levels being closely related to rumen microbiota activity and rumen fermentation (34, 35). NH₃-N is a key source of nitrogen for the synthesis of MCP by rumen microorganisms, and MCP can be a significant protein source for ruminants (36). Additionally, the heightened synthesis and supply of rumen MCP can significantly enhance milk yield and milk quality in dairy cows (37). Wang et al. (38) found that a traditional Chinese medicine compound containing *Pogostemon cablin*, Atractylodes lancea, Phellodendron chinensis, and Gypsum Fibrosum had no substantial influence on the rumen pH and NH₃-N levels after 24 h of *in vitro* ruminal fermentation. Similarly, Zhu et al. (39) demonstrated that the traditional Chinese medicine formula Yufeisan had no significant impact on rumen pH, NH₃-N, MCP, and SCFAs levels. This study found that Sijunzi San promoted the fermentation of rumen microorganisms, resulting in a downward trend in rumen pH within the normal range. Additionally, it facilitated the conversion of more non-protein nitrogen into MCP available to ruminants. The effect of Sijunzi San on ruminal fermentation performance improves feed utilization and nutrient absorption in postpartum dairy cows.

### 4.2 Regulation of Sijunzi San on rumen microbiota and SCFAs

The SCFAs were important energetic molecules for ruminants, providing 70 to 80% of the energy required by ruminants (40). Propionate acts as a gluconeogenic precursor, transformed to glucose molecules through the tricarboxylic acid cycle, and accounts for 40 to 70% of the glucose needed by ruminants (41). Acetate is capable of being absorbed via the rumen wall and subsequently transported to the liver, where it is converted to acetyl coenzyme A by enzyme catalysis and enters the tricarboxylic acid cycle for energy metabolism (42). Butyrate is able to enhance the absorption of SCFAs by promoting the proliferation of rumen epithelium and augmenting the size and surface area of papillae (43). Supplementation of the diet with propionate and butyrate significantly alleviated postpartum NEB, increased lactation, and facilitated postpartum recovery in dairy cows (44, 45). In this study, various doses of Sijunzi San increased the concentrations of propionate, acetate, and butyrate, indicating that Sijunzi San promotes rumen fermentation and enhances the production of SCFAs.

These SCFAs are produced by the complex and diverse ruminal microbial community that ferment fibrous material in the feed. Hence, the research intensified its investigation into the influence of Sijunzi San on the diversity and quantity of rumen microbiota. The findings from both alpha and beta diversity analyses clearly confirmed that Sijunzi San could enhanced the richness and evenness of rumen microbiota.

Bacteroidota and Firmicutes were the dominant phyla in the rumen of dairy cows (46). They maintain a mutually beneficial symbiotic relationship that promotes host energy absorption and storage (47, 48). Research indicated that a reduction in the relative abundance of Bacteroidota, coupled with an elevation in the relative abundance of Firmicutes, may result in gastrointestinal barrier impairment (49). Another study revealed a favorable link between *UCG-005* genus and the incidence of diarrhea (50). Sijunzi San has been reported to demonstrate excellent therapeutic effects on intestinal disorders, including loss of appetite, dyspepsia, and diarrhea (51). In this study, Sijunzi San enhanced the relative abundance of the Bacteroidota while concurrently reducing populations of the Firmicutes and the harmful bacteria genus *UCG-005*. This suggests that Sijunzi San helps sustain gastrointestinal epithelial barrier health by regulating the relative abundance of dominant rumen phyla and suppressing harmful bacterial proliferation.

The genus *Succiniclasticum* possessed the ability to swiftly transform succinic acid, a byproduct of rumen fermentation, into propionate, which serves as a precursor for glucose (52, 53). The genus *Prevotella*, a core component of the rumen microbiota, was reported to decompose hemicellulose and starch from the diet into acetate and propionate, facilitating digestion and absorption and thereby enhancing energy absorption efficiency (54, 55). This study observed that Sijunzi San significantly increased the relative abundance of *Succiniclasticum* and *Prevotella*, both of which were positively correlated with ruminal SCFAs. This suggests that Sijunzi San can enhance rumen fermentation capacity by modulating the component and abundance of rumen microbiota, thereby promoting the production of rumen SCFAs.

### 4.3 The correlation between active ingredients of Sijunzi San and rumen microbiota

Numerous active ingredients of traditional Chinese herbs have been shown to influence the health of organisms by regulating the structure and composition of the intestinal microbiota (56). Sijunzi San contains a variety of active ingredients with substantial potential to regulate rumen fermentation and microbial homeostasis (57). Bai et al. (58) found that oligosaccharides from *Codonopsis pilosula* regulated the intestinal microbiota component in a high fat diet and increased relative abundance of salutary bacterial genera for instance *Muribaculaceae*, *Alistipes*, and *Clostridium*, thereby improving obesity. Jing et al. (59) also found that the oligosaccharides could improve the relative abundance of beneficial genera *Bifidobacterium*, *Lactobacillus*, and *Akkermansia*, maintaining intestinal homeostasis in colitis model mice. This study revealed that the active compounds Luteolin and Glycitein from *Codonopsis pilosula* promote the proliferation of the genus *Prevotella*.

Xu et al. (60) demonstrated that Poria cocos was able to ameliorate antibiotic-associated diarrhea in mice by increasing the relative abundance of genera *Muribaculaceae* and *Lachnospiraceae*, decreasing harmful bacteria such as *Escherichia-Shigella* and *Staphylococcus*, and boosting the content of SCFAs. Zheng et al. (61) observed that dietary Liquorice enhanced the relative abundance of intestinal genera *Parabacteroides*, *Prevotella*, and *Bacteroidales*, improving intestinal health in cadmium-poisoned mice. Our study revealed that the main active component of Poria, Poricoic acid B, promotes the relative abundance of the genus *Christensenellaceae_R-7_group*. However, the major constituents of Liquorice, Licochalcone B and Liquoric acid, inhibit the proliferation of the genus *Comamonas*. These results collectively highlight the potential of active ingredients of Sijunzi San to modulate gut microbiota and enhance gut health.

### 4.4 Improvement of Sijunzi San on immunoglobulin levels and fat mobilization in postpartum dairy cows

Postpartum hormonal or metabolic changes in dairy cows can reduce immune function, subsequently resulting in varying degrees of immunosuppression within the organism (62). Immunosuppression in dairy cows results in decreased blood levels of immunoglobulins (IgA, IgG, and IgM), which are essential for specific immunity, and a reduction in these immunoglobulins levels indicates impaired immune function (63). Sijunzi San have been confirmed to boost the body’s immune function by elevating levels of IgA, IgG, IgM, and TNF-*α* (64, 65). Additionally, SCFAs derived from gut microbes can modulate both mucosal and systemic immune responses (66). Shipo et al. (67) observed that adding sodium acetate, propionate, or butyric acid to diets increased serum IgM levels. Biagi et al. (68) found that feeding sodium butyrate to weaned piglets could significantly increase serum IgG levels and inhibit pro-inflammatory cytokine production. The research revealed that Sijunzi San elevated blood levels of IgA, IgG, and IgM in postpartum cows, indicating that the prescription likely alleviates immunosuppression by enhancing the production of ruminal SCFAs.

In postpartum dairy cows experiencing NEB, excessive lipolysis produces substantial amounts of NEFA, which are transported to the liver through the blood circulation (69). The majority of these NEFA are utilized by the liver to generate ATP, thereby helping to compensate for energy deficits. Nevertheless, a fraction of these NEFA is converted into ketone bodies, while others are transformed into triglycerides (TG) via the actions of fatty acyl coenzyme A synthetase and glycerol kinase (70). The persistent accumulation of TG within the liver heightens the risk of postpartum cows suffering from fatty liver and ketosis (71). Furthermore, elevated blood NEFA concentrations indicate insufficient hepatic capacity to metabolize NEFA, adversely affecting health, lactation and reproductive performance of dairy cows (72). MTTP facilitates the transfer of phospholipids and TG to nascent apolipoprotein B, thereby contributing to lipoprotein formation and lipid transport (73). Studies have shown that acetate, propionate, and butyrate generated during rumen fermentation can be converted into glucose through gluconeogenesis, thereby meeting the energy demands of ruminant animals. This process effectively fills the energy deficit and reduce reliance on fat mobilization, thereby alleviating NEB in postpartum dairy cows (74). Our research revealed that Sijunzi San can markedly promote the production of ruminal SCFAs, simultaneously significantly decrease blood levels of KET, NEFA, and TG, while also increasing the blood level of MTTP in postpartum dairy cows. This suggests that Sijunzi San may slow down fat mobilization in postpartum dairy cows by promoting SCFAs production in the rumen.

### 4.5 Alteration of Sijunzi San on the blood metabolome of postpartum dairy cows

Lipid metabolism and amino acid metabolism were crucial for postpartum body recovery and milk production in dairy cows. The maintenance of their homeostatic balance is essential to ensure a continuous supply of energy and efficient nutrient utilization (75, 76). Cholesterol is vital for remaining the stability and integrity of cell membranes, but excessive accumulation can lead to atherosclerosis, hypertension, and fatty liver disease (77, 78). Luo et al. (79) found that reduction of cholesterol synthesis promotes fatty acid metabolism in hepatocytes and alleviates oxidative stress from high fatty acid load. Another study found that butyrate inhibits cholesterol excretion in the liver, thereby reducing serum cholesterol levels (80). 4-Aminobutyraldehyde is a precursor substance of *γ*-aminobutyric acid (GABA), which activates leptin receptors on neurons and reduces obesity by modulating leptin (81). Malonic semialdehyde is catalyzed by malonic semialdehyde dehydrogenase to generate acetyl coenzyme A and enters the TCA cycle to supply oxidative energy (82). Some studies have shown that Sijunzi San can promote energy production by regulating the TCA cycle (83). This suggests that Sijunzi San can aid in body recovery of postpartum dairy cows by regulating lipid metabolism and amino acid metabolism. The potential mechanisms behind this effect may be associated with the augmentation of rumen fermentation and the elevation of SCFAs production induced by Sijunzi San. The underlying mechanisms may be related to the enhancement of rumen fermentation and the enhancement of SCFAs production by Sijunzi San.

## 5 Conclusion

**Figure 12:**
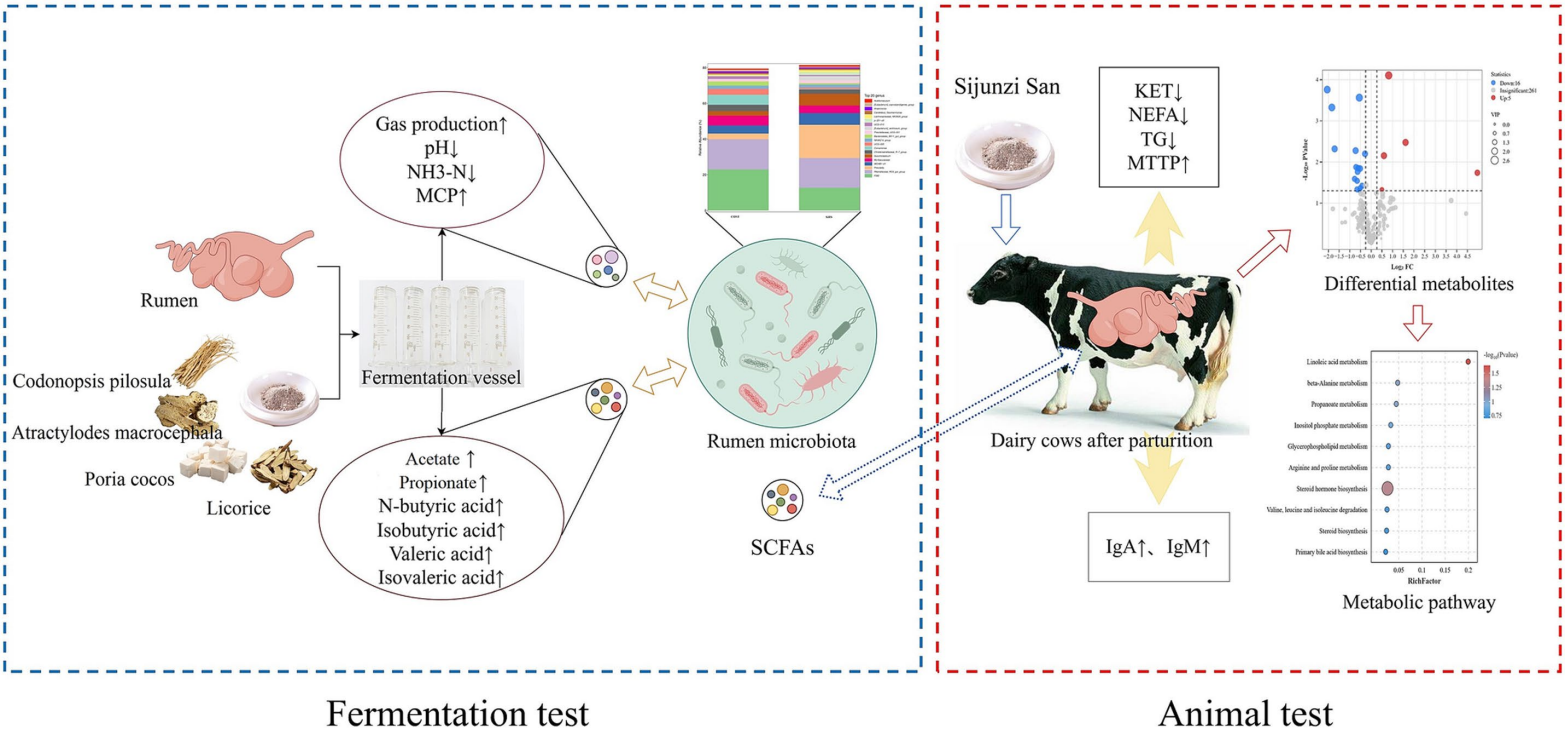
Mechanism of Sijunzi San in alleviating negative energy balance in postpartum cows by regulating the structure of rumen microbiota and fermentation capacity of postpartum cows. Created with Figdraw (https://www.figdraw.com).

This study integrated *in vitro* ruminal fermentation, gas chromatography and metabolomics to investigate the regulatory impacts of Sijunzi San on rumen microbiota, fermentation capacity and metabolism in postpartum dairy cows. The findings indicated that Sijunzi San can regulate both the composition and abundance of rumen microbiota, enhancing rumen fermentation capacity. *In vivo* results revealed that Sijunzi San can ameliorate immunosuppression, decelerate fat mobilization, and regulate lipid and amino acid metabolism in postpartum dairy cows. These outcomes suggest that Sijunzi San may alleviate NEB in postpartum dairy cows by enhancing rumen fermentation and boosting the production of ruminal SCFAs (Figure 12). The insights from this research provide novel perspectives on the pharmacological benefits of Sijunzi San in alleviating postpartum NEB in dairy cows, serving as a valuable reference for the application of Sijunzi San and related traditional Chinese veterinary medicines.

## Data Availability

The original contributions presented in the study are publicly available. This data can be found here: PRJNA1169787/https://data.mendeley.com/datasets/jhhjxgj295/1.

## Footnotes

^1^ https://www.omicshare.com/

^2^ https://www.metaboanalyst.ca/

## Supplementary material

Supplementary Figure 1 Effects of sijunzi san on blood metabolites in postpartum dairy cows. **(A)** Blood metabolite chromatogram under positive ion mode; **(B)** Blood metabolite chromatogram under negative ion mode; **(C)** Blood metabolite statistical pie chart.

Supplementary Table 1 Information table for linear regression equations for SCFAs.

Supplementary Table 2 Rumen differential microorganisms and scfas spearman correlation analysis information table.

Supplementary Table 3 Rumen differential microorganisms and scfas spearman correlation analysis information table.

## References

[1] von MAG Martin NP Kebreab E Knowlton KF Grant RJ Stephenson M. Invited review: sustainability of the us dairy industry. J Dairy Sci. (2013) 96:5405–25. doi: 10.3168/jds.2012-6354, PMID: 23831089

[2] Gross JJ Bruckmaier RM. Invited review: metabolic challenges and adaptation during different functional stages of the mammary gland in dairy cows: perspectives for sustainable milk production. J Dairy Sci. (2019) 102:2828–43. doi: 10.3168/jds.2018-15713, PMID: 30799117

[3] Mann S Yepes FA Overton TR Wakshlag JJ Lock AL Ryan CM. Dry period plane of energy: effects on feed intake, energy balance, milk production, and composition in transition dairy cows. J Dairy Sci. (2015) 98:3366–82. doi: 10.3168/jds.2014-9024 25771059

[4] Esposito G Irons PC Webb EC Chapwanya A. Interactions between negative energy balance, metabolic diseases, uterine health and immune response in transition dairy cows. Anim Reprod Sci. (2014) 144:60–71. doi: 10.1016/j.anireprosci.2013.11.007, PMID: 24378117

[5] Kuhla B Metges CC Hammon HM. Endogenous and dietary lipids influencing feed intake and energy metabolism of periparturient dairy cows. Domest Anim Endocrinol. (2016) 56:S2–S10. doi: 10.1016/j.domaniend.2015.12.002, PMID: 27345317

[6] Liu L Wu P Guo A Yang Y Chen F Zhang Q. Research progress on the regulation of production traits by gastrointestinal microbiota in dairy cows. Front Vet Sci. (2023) 10:1206346. doi: 10.3389/fvets.2023.1206346, PMID: 37592942 PMC10427726

[7] Guo Y Wang F Mao Y Kong W Wang J Zhang G. Influence of parturition on rumen bacteria and scfas in Holstein cows based on 16s rrna sequencing and targeted metabolomics. Animals (Basel). (2023) 13:782. doi: 10.3390/ani13050782 36899639 PMC10000066

[8] Gebreyesus G Difford GF Buitenhuis B Lassen J Noel SJ Højberg O. Predictive ability of host genetics and rumen microbiome for subclinical ketosis. J Dairy Sci. (2020) 103:4557–69. doi: 10.3168/jds.2019-17824, PMID: 32197852

[9] Rahman MA Redoy M Chowdhury R Al-Mamun M. Effect of dietary supplementation of plantain herb, lemongrass and their combination on milk yield, immunity, liver enzymes, serum, and milk mineral status in dairy cows. J Adv Vet Anim Res. (2024) 11:185–93. doi: 10.5455/javar.2024.k764, PMID: 38680813 PMC11055577

[10] Antonius A Pazla R Putri EM Alma’I MI Laconi EB Diapari D. Effects of herbal plant supplementation on rumen fermentation profiles and protozoan population in vitro. Vet World. (2024) 17:1139–48. doi: 10.14202/vetworld.2024.1139-1148, PMID: 38911071 PMC11188901

[11] Yi M Cao Z Zhou J Ling Y Zhang Z Cao H. Multi-omics analysis of the mechanism of mentha haplocalyx briq on the growth and metabolic regulation of fattening sheep. Animals (Basel). (2023) 13:3461. doi: 10.3390/ani13223461 38003078 PMC10668852

[12] Ma FT Shan Q Jin YH Gao D Li HY Chang MN. Effect of *lonicera japonica* extract on lactation performance, antioxidant status, and endocrine and immune function in heat-stressed mid-lactation dairy cows. J Dairy Sci. (2020) 103:10074–82. doi: 10.3168/jds.2020-18504, PMID: 32896406

[13] Ma P Peng C Peng Y Fan L Chen X Li X. A mechanism of sijunzi decoction on improving intestinal injury with spleen deficiency syndrome and the rationality of its compatibility. J Ethnopharmacol. (2023) 306:116088. doi: 10.1016/j.jep.2022.116088, PMID: 36649851

[14] Li CQ He LC Dong HY Jin JQ. Screening for the anti-inflammatory activity of fractions and compounds from atractylodes macrocephala koidz. J Ethnopharmacol. (2007) 114:212–7. doi: 10.1016/j.jep.2007.08.002, PMID: 17869038

[15] Nie A Chao Y Zhang X Jia W Zhou Z Zhu C. Phytochemistry and pharmacological activities of wolfiporia cocos (f.a. wolf) ryvarden & gilb. Front Pharmacol. (2020) 11:505249. doi: 10.3389/fphar.2020.505249, PMID: 33071776 PMC7533546

[16] Asl MN Hosseinzadeh H. Review of pharmacological effects of glycyrrhiza sp. and its bioactive compounds. Phytother Res. (2008) 22:709–24. doi: 10.1002/ptr.2362, PMID: 18446848 PMC7167813

[17] Gan D Xu A Du H Ye Y. Chinese classical formula sijunzi decoction and chronic atrophic gastritis: evidence for treatment approach? Evid Based Complement Alternat Med. (2017) 2017:9012929. doi: 10.1155/2017/9012929, PMID: 29138645 PMC5613649

[18] An K Jin-Rui G Zhen Z Xiao-Long W. Simultaneous quantification of ten active components in traditional chinese formula sijunzi decoction using a uplc-pda method. J Anal Methods Chem. (2014) 2014:570359. doi: 10.1155/2014/570359, PMID: 24963442 PMC4054979

[19] Wang Y He S Cheng X Lu Y Zou Y Zhang Q. Uplc-q-tof-ms/ms fingerprinting of traditional chinese formula sijunzitang. J Pharm Biomed Anal. (2013) 80:24–33. doi: 10.1016/j.jpba.2013.02.021, PMID: 23511229

[20] Yu S Zhao Y Li L Zhao H Liu M Jiang L. Flavonoids from citrus peel display potential synergistic effects on inhibiting rumen methanogenesis and ammoniagenesis: a microbiome perspective. Environ Sci Pollut Res Int. (2024) 31:21208–23. doi: 10.1007/s11356-024-32509-5 38383931

[21] He Z Guo J Zhang H Yu J Zhou Y Wang Y. Atractylodes macrocephala koidz polysaccharide improves glycolipid metabolism disorders through activation of aryl hydrocarbon receptor by gut flora-produced tryptophan metabolites. Int J Biol Macromol. (2023) 253:126987. doi: 10.1016/j.ijbiomac.2023.126987, PMID: 37729987

[22] Menke KH Raab L Salewski A Steingass H Fritz D Schneider W. The estimation of the digestibility and metabolizable energy content of ruminant feedingstuffs from the gas production when they are incubated with rumen liquorin vitro. J Agric Sci. (1979) 93:217–22. doi: 10.1017/S0021859600086305

[23] Broderick GA Kang JH. Automated simultaneous determination of ammonia and total amino acids in ruminal fluid and in vitro media. J Dairy Sci. (1980) 63:64–75. doi: 10.3168/jds.S0022-0302(80)82888-8, PMID: 7372898

[24] Makkar HP Sharma OP Dawra RK Negi SS. Simple determination of microbial protein in rumen liquor. J Dairy Sci. (1982) 65:2170–3. doi: 10.3168/jds.S0022-0302(82)82477-6, PMID: 7153399

[25] Blaxter M Mann J Chapman T Thomas F Whitton C Floyd R. Defining operational taxonomic units using dna barcode data. Philos Trans R Soc Lond Ser B Biol Sci. (2005) 360:1935–43. doi: 10.1098/rstb.2005.1725, PMID: 16214751 PMC1609233

[26] Anderson MJ Ellingsen KE Mcardle BH. Multivariate dispersion as a measure of beta diversity. Ecol Lett. (2006) 9:683–93. doi: 10.1111/j.1461-0248.2006.00926.x, PMID: 16706913

[27] Ramette A. Multivariate analyses in microbial ecology. FEMS Microbiol Ecol. (2007) 62:142–60. doi: 10.1111/j.1574-6941.2007.00375.x 17892477 PMC2121141

[28] Menezes EB Velho A Santos F Dinh T Kaya A Topper E. Uncovering sperm metabolome to discover biomarkers for bull fertility. BMC Genomics. (2019) 20:714. doi: 10.1186/s12864-019-6074-6, PMID: 31533629 PMC6749656

[29] Kanehisa M Furumichi M Tanabe M Sato Y Morishima K. Kegg: new perspectives on genomes, pathways, diseases and drugs. Nucleic Acids Res. (2017) 45:D353–61. doi: 10.1093/nar/gkw1092, PMID: 27899662 PMC5210567

[30] Waterman RC Richardson KD Lodge-Ivey SL. Effects of euphorbia esula l. (leafy spurge) on cattle and sheep in vitro fermentation and gas production. J Sci Food Agric. (2011) 91:2053–60. doi: 10.1002/jsfa.4419, PMID: 21520450

[31] Amanzougarene Z Fondevila M. Fitting of the in vitro gas production technique to the study of high concentrate diets. Animals (Basel). (2020) 10:1935. doi: 10.3390/ani10101935 33096765 PMC7590040

[32] Antonius A Pazla R Putri EM Negara W Laia N Ridla M. Effectiveness of herbal plants on rumen fermentation, methane gas emissions, in vitro nutrient digestibility, and population of protozoa. Vet World. (2023) 16:1477–88. doi: 10.14202/vetworld.2023.1477-1488, PMID: 37621549 PMC10446706

[33] Peng Z Fujino M Anand M Uyeno Y. Feeding astragalus membranaceus root improves the rumen fermentation rate in housed goats through the alteration of the rumen community composition. Microorganisms. (2024) 12:1067. doi: 10.3390/microorganisms12061067, PMID: 38930452 PMC11205705

[34] Deusch S Camarinha-Silva A Conrad J Beifuss U Rodehutscord M Seifert J. A structural and functional elucidation of the rumen microbiome influenced by various diets and microenvironments. Front Microbiol. (2017) 8:1605. doi: 10.3389/fmicb.2017.01605, PMID: 28883813 PMC5573736

[35] Wang Y Wang L Wang Z Xue B Peng Q Hu R. Recent advances in research in the rumen bloat of ruminant animals fed high-concentrate diets. Front Vet Sci. (2023) 10:1142965. doi: 10.3389/fvets.2023.1142965 37035805 PMC10076780

[36] Claassen RM Christensen DA Mutsvangwa T. Effects of extruding wheat dried distillers grains with solubles with peas or canola meal on ruminal fermentation, microbial protein synthesis, nutrient digestion, and milk production in dairy cows. J Dairy Sci. (2016) 99:7143–58. doi: 10.3168/jds.2015-10808, PMID: 27394944

[37] Zhu W Fu Y Wang B Wang C Ye JA Wu YM. Effects of dietary forage sources on rumen microbial protein synthesis and milk performance in early lactating dairy cows. J Dairy Sci. (2013) 96:1727–34. doi: 10.3168/jds.2012-5756, PMID: 23295118

[38] Wang SP Wang WJ Tan ZL Liu GW Zhou CF Yin MJ. Effect of traditional chinese medicine compounds on rumen fermentation, methanogenesis and microbial flora in vitro. Anim Nutr. (2019) 5:185–90. doi: 10.1016/j.aninu.2018.09.004 31193871 PMC6544579

[39] Zhu Z Song ZH Cao LT Wang Y Zhou WZ Zhou P. Effects of traditional chinese medicine formula on ruminal fermentation, enzyme activities and nutrient digestibility of beef cattle. Anim Sci J. (2018) 89:661–71. doi: 10.1111/asj.12978, PMID: 29327395

[40] Mirzaei R Dehkhodaie E Bouzari B Rahimi M Gholestani A Hosseini-Fard SR. Dual role of microbiota-derived short-chain fatty acids on host and pathogen. Biomed Pharmacother. (2022) 145:112352. doi: 10.1016/j.biopha.2021.112352, PMID: 34840032

[41] Louis P Flint HJ. Formation of propionate and butyrate by the human colonic microbiota. Environ Microbiol. (2017) 19:29–41. doi: 10.1111/1462-2920.13589 27928878

[42] Kindt A Liebisch G Clavel T Haller D Hormannsperger G Yoon H. The gut microbiota promotes hepatic fatty acid desaturation and elongation in mice. Nat Commun. (2018) 9:3760. doi: 10.1038/s41467-018-05767-4, PMID: 30218046 PMC6138742

[43] Liu L Sun D Mao S Zhu W Liu J. Infusion of sodium butyrate promotes rumen papillae growth and enhances expression of genes related to rumen epithelial vfa uptake and metabolism in neonatal twin lambs. J Anim Sci. (2019) 97:909–21. doi: 10.1093/jas/sky459, PMID: 30535158 PMC6377441

[44] Zhang F Zhao Y Wang H Nan X Wang Y Guo Y. Alterations in the milk metabolome of dairy cows supplemented with different levels of calcium propionate in early lactation. Meta. (2022) 12:699. doi: 10.3390/metabo12080699, PMID: 36005569 PMC9415114

[45] Zhang J Bu L Liu Y Huo W Xia C Pei C. Dietary supplementation of sodium butyrate enhances lactation performance by promoting nutrient digestion and mammary gland development in dairy cows. Anim Nutr. (2023) 15:137–48. doi: 10.1016/j.aninu.2023.08.008, PMID: 38023376 PMC10661553

[46] Mao S Zhang M Liu J Zhu W. Characterising the bacterial microbiota across the gastrointestinal tracts of dairy cattle: membership and potential function. Sci Rep. (2015) 5:16116. doi: 10.1038/srep16116, PMID: 26527325 PMC4630781

[47] Lilian M Rawlynce B Charles G Felix K. Potential role of rumen bacteria in modulating milk production and composition of admixed dairy cows. Lett Appl Microbiol. (2023) 76:ovad007. doi: 10.1093/lambio/ovad007 36722031

[48] Huuki H Ahvenjarvi S Lidauer P Popova M Vilkki J Vanhatalo A. Fresh rumen liquid inoculant enhances the rumen microbial community establishment in pre-weaned dairy calves. Front Microbiol. (2021) 12:758395. doi: 10.3389/fmicb.2021.758395, PMID: 35095788 PMC8790516

[49] Li X Wang L. Highland barley attenuates high fat and cholesterol diet induced hyperlipidemia in mice revealed by 16s rrna gene sequencing and untargeted metabolomics. Life Sci. (2023) 334:122142. doi: 10.1016/j.lfs.2023.122142, PMID: 37797689

[50] Liang J Kou S Chen C Raza S Wang S Ma X. Effects of *clostridium butyricum* on growth performance, metabonomics and intestinal microbial differences of weaned piglets. BMC Microbiol. (2021) 21:85. doi: 10.1186/s12866-021-02143-z, PMID: 33752593 PMC7983215

[51] Li D Wang Y Liu N Chen S Liu H Wang P. Modified sijunzi granule decreases post-weaning diarrhea in rex rabbits via promoting intestinal development. Front Vet Sci. (2022) 9:972326. doi: 10.3389/fvets.2022.972326, PMID: 36419729 PMC9676230

[52] van Gylswyk NO. Succiniclasticum ruminis gen. nov., sp. nov., a ruminal bacterium converting succinate to propionate as the sole energy-yielding mechanism. Int J Syst Bacteriol. (1995) 45:297–300. doi: 10.1099/00207713-45-2-297, PMID: 7537062

[53] Mcloughlin S Spillane C Campion FP Claffey N Sosa CC Mcnicholas Y. Breed and ruminal fraction effects on bacterial and archaeal community composition in sheep. Sci Rep. (2023) 13:3336. doi: 10.1038/s41598-023-28909-1, PMID: 36849493 PMC9971215

[54] Holman DB Gzyl KE. A meta-analysis of the bovine gastrointestinal tract microbiota. FEMS Microbiol Ecol. (2019) 95:fiz072. doi: 10.1093/femsec/fiz072 31116403

[55] Fan Q Wanapat M Yan T Hou F. Altitude influences microbial diversity and herbage fermentation in the rumen of yaks. BMC Microbiol. (2020) 20:370. doi: 10.1186/s12866-020-02054-5, PMID: 33276718 PMC7718673

[56] Gong X Li X Bo A Shi RY Li QY Lei LJ. The interactions between gut microbiota and bioactive ingredients of traditional chinese medicines: a review. Pharmacol Res. (2020) 157:104824. doi: 10.1016/j.phrs.2020.104824 32344049

[57] Wu Y Zheng Y Wang X Tang P Guo W Ma H. Ginseng-containing sijunzi decoction ameliorates ulcerative colitis by orchestrating gut homeostasis in microbial modulation and intestinal barrier integrity. Am J Chin Med. (2023) 51:677–99. doi: 10.1142/S0192415X23500325, PMID: 36883990

[58] Bai R Cui F Li W Wang Y Wang Z Gao Y. *Codonopsis pilosula* oligosaccharides modulate the gut microbiota and change serum metabolomic profiles in high-fat diet-induced obese mice. Food Funct. (2022) 13:8143–57. doi: 10.1039/d2fo01119k, PMID: 35816111

[59] Jing Y Li A Liu Z Yang P Wei J Chen X. Absorption of *codonopsis pilosula* saponins by coexisting polysaccharides alleviates gut microbial dysbiosis with dextran sulfate sodium-induced colitis in model mice. Biomed Res Int. (2018) 2018:1–18. doi: 10.1155/2018/1781036 PMC6120299 30211217

[60] Xu H Wang S Jiang Y Wu J Chen L Ding Y. Poria cocos polysaccharide ameliorated antibiotic-associated diarrhea in mice via regulating the homeostasis of the gut microbiota and intestinal mucosal barrier. Int J Mol Sci. (2023) 24:1423. doi: 10.3390/ijms24021423 36674937 PMC9862632

[61] Zheng X Wang L You L Liu YX Cohen M Tian S. Dietary licorice enhances in vivo cadmium detoxification and modulates gut microbial metabolism in mice. iMeta. (2022) 1:e7. doi: 10.1002/imt2.7, PMID: 38867726 PMC10989944

[62] Moyes KM Drackley JK Salak-Johnson JL Morin DE Hope JC Loor JJ. Dietary-induced negative energy balance has minimal effects on innate immunity during a *streptococcus uberis* mastitis challenge in dairy cows during midlactation. J Dairy Sci. (2009) 92:4301–16. doi: 10.3168/jds.2009-2170, PMID: 19700690

[63] Aleri JW Hine BC Pyman MF Mansell PD Wales WJ Mallard B. Periparturient immunosuppression and strategies to improve dairy cow health during the periparturient period. Res Vet Sci. (2016) 108:8–17. doi: 10.1016/j.rvsc.2016.07.007, PMID: 27663364

[64] Chen JM Yang TT Cheng TS Hsiao TF Chang PM Leu JY. Modified sijunzi decoction can alleviate cisplatin-induced toxicity and prolong the survival time of cachectic mice by recovering muscle atrophy. J Ethnopharmacol. (2019) 233:47–55. doi: 10.1016/j.jep.2018.12.035, PMID: 30590199

[65] Liang H Guo J Li CG. Long-term complete remission of a patient with double-hit diffuse large b-cell lymphoma treated by chemoimmunotherapy and chinese herbal medicine. Integr Cancer Ther. (2023) 22:1553462651. doi: 10.1177/15347354221147515 PMC9900160 36722702

[66] Mann ER Lam YK Uhlig HH. Short-chain fatty acids: linking diet, the microbiome and immunity. Nat Rev Immunol. (2024) 24:577–95. doi: 10.1038/s41577-024-01014-8, PMID: 38565643

[67] Li S Heng X Guo L Lessing DJ Chu W. Scfas improve disease resistance via modulate gut microbiota, enhance immune response and increase antioxidative capacity in the host. Fish Shellfish Immunol. (2022) 120:560–8. doi: 10.1016/j.fsi.2021.12.035, PMID: 34958920

[68] Biagi G Piva A Moschini M Vezzali E Roth FX. Performance, intestinal microflora, and wall morphology of weanling pigs fed sodium butyrate. J Anim Sci. (2007) 85:1184–91. doi: 10.2527/jas.2006-378, PMID: 17296766

[69] Ringseis R Gessner DK Eder K. Molecular insights into the mechanisms of liver-associated diseases in early-lactating dairy cows: hypothetical role of endoplasmic reticulum stress. J Anim Physiol Anim Nutr (Berl). (2015) 99:626–45. doi: 10.1111/jpn.12263, PMID: 25319457

[70] Tamminga S. The effect of the supply of rumen degradable protein and metabolisable protein on negative energy balance and fertility in dairy cows. Anim Reprod Sci. (2006) 96:227–39. doi: 10.1016/j.anireprosci.2006.08.003, PMID: 16979310

[71] Itle AJ Huzzey JM Weary DM von Keyserlingk MA. Clinical ketosis and standing behavior in transition cows. J Dairy Sci. (2015) 98:128–34. doi: 10.3168/jds.2014-7932, PMID: 25465623

[72] Sun F Cao Y Cai C Li S Yu C Yao J. Regulation of nutritional metabolism in transition dairy cows: energy homeostasis and health in response to post-ruminal choline and methionine. PLoS One. (2016) 11:e160659. doi: 10.1371/journal.pone.0160659, PMID: 27501393 PMC4976856

[73] Hooper AJ Burnett JR Watts GF. Contemporary aspects of the biology and therapeutic regulation of the microsomal triglyceride transfer protein. Circ Res. (2015) 116:193–205. doi: 10.1161/CIRCRESAHA.116.304637, PMID: 25552696

[74] Aschenbach JR Kristensen NB Donkin SS Hammon HM Penner GB. Gluconeogenesis in dairy cows: the secret of making sweet milk from sour dough. IUBMB Life. (2010) 62:869–77. doi: 10.1002/iub.400, PMID: 21171012

[75] Mcfadden JW. Review: lipid biology in the periparturient dairy cow: contemporary perspectives. Animal. (2020) 14:s165–75. doi: 10.1017/S1751731119003185 32024571

[76] Zhao Z Dong J Wang D Zhao C Tian X Meng Y. Metabolomic analysis of rumen-protected branched-chain amino acids in primiparous dairy cows. Front Immunol. (2024) 15:1385896. doi: 10.3389/fimmu.2024.1385896, PMID: 38715606 PMC11075066

[77] Jaureguiberry MS Tricerri MA Sanchez SA Garda HA Finarelli GS Gonzalez MC. Membrane organization and regulation of cellular cholesterol homeostasis. J Membr Biol. (2010) 234:183–94. doi: 10.1007/s00232-010-9245-6 20336284 PMC2868589

[78] Guo J Chen S Zhang Y Liu J Jiang L Hu L. Cholesterol metabolism: physiological regulation and diseases. MedComm (2020). (2024) 5:e476. doi: 10.1002/mco2.476 38405060 PMC10893558

[79] Luo J Yang H Song BL. Mechanisms and regulation of cholesterol homeostasis. Nat Rev Mol Cell Biol. (2020) 21:225–45. doi: 10.1038/s41580-019-0190-7 31848472

[80] Ye X Shen S Xu Z Zhuang Q Xu J Wang J. Sodium butyrate alleviates cholesterol gallstones by regulating bile acid metabolism. Eur J Pharmacol. (2021) 908:174341. doi: 10.1016/j.ejphar.2021.174341, PMID: 34273384

[81] Liao Y Wang C Gao Z Pan Z Peng M Ma J. Anti-obesity mechanism of ganpu tea revealed by microbiome, metabolome and transcriptome analyses. Food Chem. (2023) 412:135048. doi: 10.1016/j.foodchem.2022.135048, PMID: 36753939

[82] Jakoby WB Yamada EW. Direct enzymic conversion of malonic semialdehyde to acetyl-coenzyme a. Biochim Biophys Acta. (1959) 34:276–7. doi: 10.1016/0006-3002(59)90269-0, PMID: 13853085

[83] Dai L Liu Z Zhou W Zhang L Miao M Wang L. Sijunzi decoction, a classical chinese herbal formula, improves fatigue symptoms with changes in gut microbiota in chronic fatigue syndrome: a randomized, double-blind, placebo-controlled, multi-center clinical trial. Phytomedicine. (2024) 129:155636. doi: 10.1016/j.phymed.2024.155636, PMID: 38640860

