# Supplementary figures and images for: Sijunzi San alleviates the negative energy balance in postpartum dairy cows by regulating rumen fermentation capacity
Source: Front Vet Sci. 2024 Dec 18;11:1512081. doi: 10.3389/fvets.2024.1512081 (PMC11688294; doi:10.3389/fvets.2024.1512081)

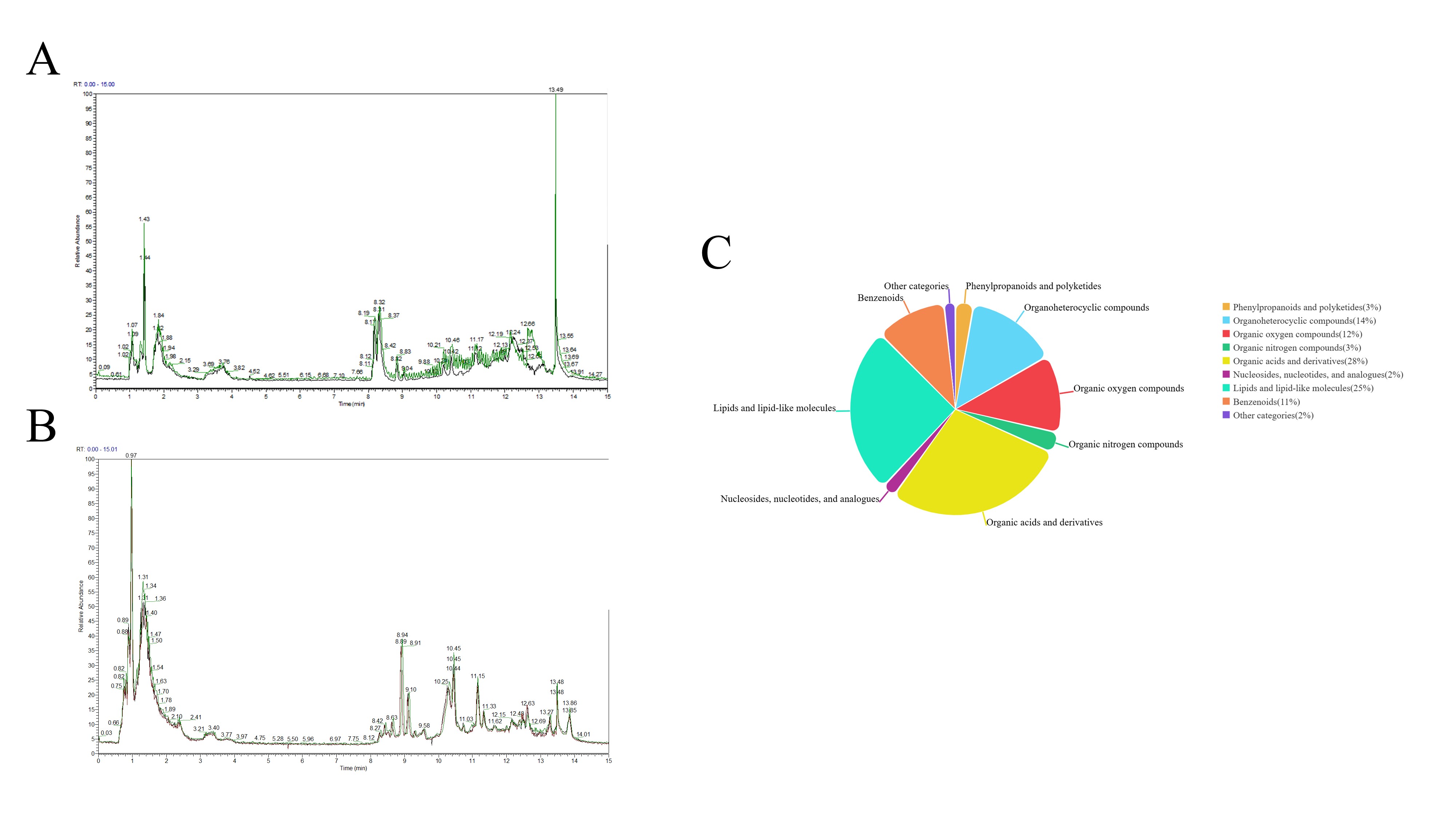

Supplement: Supplementary Figure 1 — Effects of sijunzi san on blood metabolites in postpartum dairy cows. (A) Blood metabolite chromatogram under positive ion mode; (B) Blood metabolite chromatogram under negative ion mode; (C) Blood metabolite statistical pie chart. [file Image_1.JPEG]
