# Supplementary material for: Sijunzi San alleviates the negative energy balance in postpartum dairy cows by regulating rumen fermentation capacity
Source: Front Vet Sci. 2024 Dec 18;11:1512081. doi: 10.3389/fvets.2024.1512081 (PMC11688294; doi:10.3389/fvets.2024.1512081)
Supplement: Supplementary Table 1 — Information table for linear regression equations for SCFAs. [file Table_1.DOCX]

Supplementary Table S1 Information table for linear regression equations for SCFAs

| Component | Retention Time (min) | Equation | R |
| --- | --- | --- | --- |
| Acetate | 2.598 | y =13621.2* x + 7627.47 | 0.988 |
| Propionate | 3.168 | y = 21560.2 * x + 5898.78 | 0.993 |
| Isobutyric acid | 3.350 | y = 34442.7 * x + 4097.11 | 0.988 |
| N-butyric acid | 3.842 | y = 30191.6 * x+ 9869.68 | 0.994 |
| Isovaleric acid | 4.155 | y = 39200.4 * x + 2177.58 | 0.995 |
| N-valeric acid | 4.733 | y = 37176.9 * x + 6648.87 | 0.993 |
